# PbSnRK1‐Mediated Phosphorylation of PbTCP13 Regulates Glucose‐Responsive Lignin Biosynthesis via PbMYB11 During Pear Stone Cell Formation

**DOI:** 10.1111/pbi.70770

**Published:** 2026-10-15

**Authors:** Jianan He, Xuxu Wang, Abdul Basit, Wen Li, Jiahui Chao, Wenyu Zhao, Mingxu Niu, Xin Yan, Yuanhui Yu, Kaijie Qi, Zhihua Xie, Shaoling Zhang, Katsuhiro Shiratake, Khanizadeh Shahrokh, Shutian Tao

**Affiliations:** ^1^ Sanya Institute, State Key Laboratory of Crop Genetics and Germplasm Enhancement, College of Horticulture Nanjing Agricultural University Nanjing China; ^2^ State Key Laboratory of Ecological Safety and Sustainable Development in Arid Lands, Xinjiang Institute of Ecology and Geography Chinese Academy of Sciences Urumqi China; ^3^ Laboratory of Horticultural Science, Graduate School of Bioagricultural Sciences Nagoya University Nagoya Japan; ^4^ Elm Consulting Genetic Research St‐Lazare Quebec Canada

## Abstract

Stone cell lignification is a key determinant of pear fruit texture and commercial quality, and this process has been reported to be tightly coupled to cellular glucose availability. However, how glucose dose‐dependently targets transcription factor phosphorylation during lignin biosynthesis remains largely unknown. Using yeast two‐hybrid library screening and phosphorylation assays, we found that PbSnRK1, a glucose‐responsive kinase, interacts with and directly phosphorylates PbTCP13, thereby participating in the regulation of stone cell lignification. PbSnRK1 serves as an energy‐status sensor linking glucose signalling to biphasic regulation of lignin metabolism in pear. Under low‐glucose conditions, activated PbSnRK1 phosphorylates the transcription factor PbTCP13 at Ser265, targeting it for 26S proteasome‐mediated degradation and thereby derepressing PbMYB11‐*PbCCR1* cascade associated with lignin biosynthesis. Under high‐glucose conditions, suppressed PbSnRK1 kinase activity results in PbTCP13 protein accumulation, which restricts excessive lignification by both repressing *PbCCR1* transcription and physically interacting with PbMYB11 to inhibit its transactivation capacity. This PbSnRK1‐PbTCP13‐PbMYB11 cascade, balanced by glucose concentration, acts as a molecular switch that activates stone cell formation and lignification at low‐glucose levels during early fruit development, while restricting excessive lignification at high‐glucose levels during later stages, providing new mechanistic insights into energy‐coupled secondary wall metabolism in fleshy fruits.

## 1 Introduction

Pear (*Pyrus*) is one of the most economically important temperate fruit crops worldwide. Asian pears, which account for approximately 70% of global pear production, are characterized by exceptionally high stone cell content in the flesh, a trait far more pronounced than in most other fleshy fruits. These sclereids originate from parenchyma cells through progressive secondary cell wall thickening and massive lignin deposition (Tao et al. 2009; Gong et al. 2023). As the predominant component of stone cell secondary cell walls, lignin imparts the sclerotic rigidity responsible for the characteristic gritty texture that adversely affects consumer acceptance and processing suitability (Jin et al. 2013). Therefore, elucidating the regulatory mechanisms underlying lignin biosynthesis is central to the genetic improvement of pear fruit quality. Lignin biosynthesis is a tightly regulated process that integrates developmental programmes with environmental and metabolic cues, among which sugars serve a dual role as both essential carbon precursors and central signalling molecules coordinating plant growth and metabolism (Rolland et al. 2006; Lastdrager et al. 2014).

In pear fruit, stone cell content is negatively correlated with soluble sugar levels, establishing a carbon allocation trade‐off between lignin deposition and sugar accumulation that directly impacts fruit quality (Liu et al. 2021). Glucose lies at the nexus of this trade‐off. As the primary direct carbon source for lignin, glucose metabolites are channelled through glycolysis and the phenylpropanoid pathway to supply monolignol precursors (Morandi et al. 2008). Beyond this nutritional function, glucose also provides signalling information that governs the timing and location of lignin deposition (Wang et al. 2021; Jiao et al. 2022; Sun et al. 2018). Consistent with this dual role, exogenous glucose has been shown to promote lignin accumulation in a dose‐dependent manner in both pear calli and grapevine stems (Jiao et al. 2022; Wang, Huang, et al. 2024; Wang, Wang, et al. 2024). Notably, these concentration‐dependent effects cannot be fully explained by increased carbon substrate supply alone, as equivalent carbon inputs from other sugar sources fail to reproduce the same regulatory magnitude, confirming that glucose functions as a bona fide signalling molecule governing lignin biosynthesis. However, the molecular mechanisms that perceive and transduce glucose signals to mediate concentration‐dependent lignin deposition during pear stone cell development remain largely unknown.

The evolutionarily conserved kinase SnRK1 functions as a core cellular energy sensor that translates glucose availability into adaptive metabolic reprogramming (Baena‐González et al. 2007; Robaglia et al. 2012). Its activity is tightly coupled to cellular sugar status: it is activated under energy limitation and suppressed under high‐glucose conditions, making it a quantitative sensor of cellular energy state (Baena‐González et al. 2007; Cho et al. 2012). This antagonistic regulatory pattern is evolutionarily conserved across plant species (Wang et al. 2019). Beyond its role in primary metabolism, SnRK1 directly regulates lignin biosynthesis through phosphorylation of specific transcription factors (TFs). In cassava, MeKIN10 (SnRK1α) phosphorylates MeHB16 to promote lignin deposition (Yan et al. 2024). In tree peony, PoKIN10 (SnRK1α) phosphorylates PoWRKY31 to enhance lignin biosynthesis (Luan et al. 2025). However, the downstream phosphorylation substrates and regulatory mechanisms of SnRK1 in fruit crop lignin biosynthesis remain largely unknown.

Lignin biosynthesis is governed by a hierarchical transcriptional regulatory network. Multiple TFs, including NAC, MYB, MADS, WRKY and HD‐Zip, collectively coordinate secondary cell wall formation and stone cell development (Xue et al. 2019, 2023, 2026; Gong et al. 2024; Wang, Huang, et al. 2024; Wang, Wang, et al. 2024; Luan et al. 2025). Among these, R2R3‐MYB TFs constitute the core regulatory layer, directly binding to AC‐rich cis‐elements in the promoters of lignin biosynthetic structural genes to activate their expression (Zhong et al. 2008). In pear, *PbrMYB169*, *PbrMYB24* and *PbrMYB73* function as transcriptional activators that promote lignin biosynthesis during stone cell development (Xue et al. 2019, 2023; Gong et al. 2024). Beyond the established MYB regulators, TCP TFs have recently emerged as important contributors to lignin and secondary cell wall regulation across species. In Arabidopsis, *TCP4* directly activates *VND7* to trigger a transcriptional cascade associated with secondary cell wall biosynthesis (Sun et al. 2017); in poplar, *TCP23* negatively regulates lignin deposition through the TCP23‐WRKY15 module (Zhang et al. 2025); and in cotton, *GbTCP20* regulates lignin deposition to confer wilt resistance (Wang et al. 2025). In pear, *PbTCP14* and *PbTCP15* exhibit expression patterns that closely mirror lignin accumulation and stone cell content during fruit development (Zhao et al. 2021). Notably, TCP proteins frequently serve as integrators of developmental and metabolic signals, and their activities are subject to post‐translational regulation (Hou et al. 2020; Xu et al. 2024). However, whether TCP proteins function as phosphorylation substrates of SnRK1 to transduce glucose signals into the transcriptional control of lignin biosynthesis, and how they cooperate with MYB regulators during pear stone cell development, remain to be elucidated.

Here, we identify a glucose‐responsive PbSnRK1‐PbTCP13‐PbMYB11 regulatory cascade that mediates concentration‐dependent lignin biosynthesis during pear stone cell development. We demonstrate that the energy sensor PbSnRK1 modulates the protein stability and transcriptional repressive activity of PbTCP13 via phosphorylation, which in turn releases the core lignin activator PbMYB11 to drive lignin biosynthetic gene expression. These findings improve our understanding of the mechanistic linkage between cellular carbon status and secondary cell wall metabolism in fleshy fruits and identify candidate genetic targets for improving pear fruit texture through molecular breeding.

## 2 Results

### 2.1 Glucose Promotes Lignin Biosynthesis in Pear via the PbSnRK1 Kinase

To investigate the effect of glucose on lignin formation, pear fruit calli were treated with a gradient of glucose concentrations (0%, 1%, 2%, 3%, 4%, 6%, w/v) for 14 days. Lignin content increased in a dose‐dependent manner, peaking at 4% glucose (Figure S1a,b), consistent with previous studies (Jiao et al. 2022). In accordance with the lignin accumulation pattern, the expression levels of key lignin biosynthetic genes (*PbC3H1*, *PbC4H1*, *PbCCoAOMT*, *PbCCR1*, *PbCCR2*, *PbCSE1*, *PbHCT17* and *PbPOD1*) were significantly upregulated with increasing glucose concentrations and also reached the highest level at 4% glucose (Figure S1c–j). To exclude the possibility that this effect was attributable to osmotic stress rather than glucose signalling, calli were treated with equiosmotic mannitol. Notably, 4% glucose significantly promoted lignin accumulation and upregulated the expression of key lignin biosynthetic genes compared with mannitol treatment (Figure S1k–t). These results demonstrate that glucose promotes lignin biosynthesis through a specific signalling mechanism rather than osmotic effects.

To identify the glucose signalling components involved in this process, previously published transcriptome datasets from 0% (control) and 4% glucose‐treated pear calli (Jiao et al. 2022; NCBI BioProject accession: PRJNA801923) were analysed. Among the three major glucose sensors in higher plants‐HXK1, RGS1 and SnRK1 (Li and Sheen 2016), *PbSnRK1* showed the strongest negative correlation with lignin accumulation based on transcript abundance (FPKM), whereas *PbHXK1* and *PbRGS1* showed no significant correlation (Figure 1a; Table S3). We further verified the expression pattern of *PbSnRK1* under the glucose gradient. The results showed that *PbSnRK1* expression was significantly downregulated in a dose‐dependent manner (Figure S2a), suggesting feedback inhibition of SnRK1 signalling under high‐energy conditions. Consistently, treatment with the SnRK1‐specific inhibitor trehalose‐6‐phosphate (T6P) (Blanford et al. 2024) largely abolished glucose‐induced lignin accumulation and the upregulation of lignin biosynthetic genes (Figure S2b–k). Collectively, these results indicate that glucose promotes lignin biosynthesis in pear fruit calli primarily through the *PbSnRK1*‐mediated signalling pathway.

**FIGURE 1:**
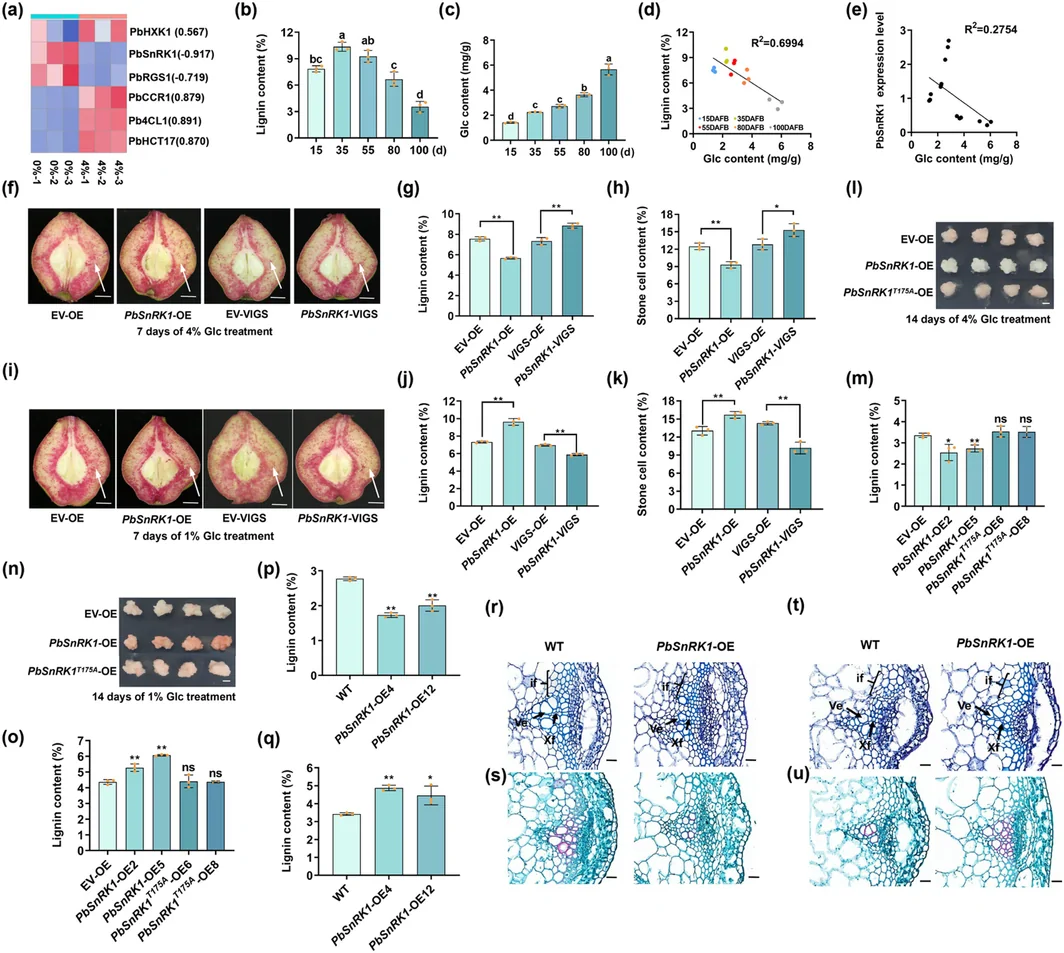
*PbSnRK1* mediates glucose‐dependent bidirectional regulation of lignin biosynthesis. (a) RNA‐seq expression profiles of sugar signalling genes and lignin biosynthetic genes and their correlation with lignin content, based on published pear calli transcriptomes (Jiao et al. 2022). (b, c) Developmental dynamics of (b) lignin content and (c) glucose content in ‘Suli’ pear fruit. (d) Correlation between glucose and lignin contents across ‘Suli’ fruit development. Dots in different colours represent distinct developmental stages. (e) Correlation between endogenous glucose content and relative transcript level of *PbSnRK1* during ‘Suli’ fruit development. (f) Phloroglucinol‐HCl staining of EV‐OE, *PbSnRK1*‐OE, EV‐VIGS and *PbSnRK1*‐VIGS transgenic pear fruits following 4% glucose treatment (scale bar, 0.5 cm). (g) Lignin content and (h) stone cell content in the above transgenic pear fruits. (i) Phloroglucinol‐HCl staining of the above transgenic pear fruits following 1% glucose treatment (scale bar, 0.5 cm). (j) Lignin content and (k) stone cell content in the above transgenic pear fruits. (l,n) Phloroglucinol‐HCl staining of EV‐OE, *PbSnRK1*‐OE and *PbSnRK1* ^*T175A*^‐OE transgenic pear calli under (l) 4% or (n) 1% glucose treatment (scale bar, 0.5 cm). (m,o) Lignin content under (m) 4% or (o) 1% glucose treatment. (p, q) Lignin content of *PbSnRK1‐*overexpressing Arabidopsis under (p) 4% or (q) 1% glucose treatment. (r‐u) Histochemical staining of cross‐sections from inflorescence stems of WT and *PbSnRK1*‐OE Arabidopsis (scale bar, 25 μm). (r) Toluidine blue O staining under 4% or (t) 1% glucose treatment; (s) safranin O‐fast green staining under 4% or (u) 1% glucose treatment. ve, vessel; xf, xylary fibre; if, interfascicular fibre. Data are presented as means ± SE (*n* = 3). Statistical significance was determined by two‐tailed Student's *t*‐test (n.s., no significance, *p* > 0.05; *p < 0.05; **p < 0.01). Different lowercase letters indicate significant differences determined by one‐way ANOVA (*p* < 0.05).

### 2.2 *PbSnRK1* Mediates Glucose‐Dependent Bidirectional Regulation of Lignin Biosynthesis

To explore the endogenous coupling between glucose availability and stone cell lignification in pear fruit, the developmental dynamics of lignin, stone cell, and glucose contents were first profiled across 14 pear cultivars with contrasting stone cell phenotypes. Lignin and stone cell contents both displayed a conserved unimodal pattern across all cultivars, increasing rapidly at early stages and declining towards fruit maturation. High‐stone cell cultivars consistently had higher lignin and stone cell levels throughout development relative to low‐stone cell cultivars (Figure S3a,b). By contrast, glucose content accumulated continuously along with fruit development in all cultivars, showing an opposite temporal trajectory to lignin deposition (Figure S3c). We then assessed the correlation between glucose and lignin contents across the full population, as well as stratified into high‐stone cell and low‐stone cell subgroups. Glucose content was significantly and negatively correlated with lignin content in the combined population and both separate subgroups (Figure S3d–f). The proportion of lignin variation explained by glucose content was higher in the high‐stone cell group (*R* ^2^ = 0.4544) than in the whole population (*R* ^2^ = 0.3915) and the low‐stone cell group (*R* ^2^ = 0.2898) (Figure S3d–f). Extending the correlation analysis to other major soluble sugars showed that the negative association with lignin content was strongest for glucose (Figure S3d), weaker for fructose (*R* ^2^ = 0.3343) and sorbitol (*R* ^2^ = 0.0993), and negligible and non‐significant for sucrose (*R* ^2^ = 0.0107) (Figure S3g–i), supporting the focus on glucose as the relevant sugar signal. We further characterized the developmental dynamics in ‘Suli’ pear across five growth stages. Lignin content showed a typical unimodal pattern, peaking at 35 days after full bloom (DAFB) and declining gradually towards maturation (Figure 1b), while glucose content increased continuously throughout development (Figure 1c). Linear regression verified a strong negative correlation between glucose and lignin across the whole developmental period (Figure 1d, *R* ^2^ = 0.6994). We also examined the association between endogenous glucose content and transcript levels of *PbSnRK1*. *PbSnRK1* expression was negatively correlated with glucose content during fruit development (Figure 1e, *R* ^2^ = 0.2754), with its expression peak coinciding with the rapid lignin accumulation phase at early development. These in vivo correlative results indicate that glucose is tightly coupled to stone cell lignification in pear fruit, and *PbSnRK1* may act as a glucose‐responsive mediator in this process.

To determine whether *PbSnRK1* functions as a glucose sensor regulating lignin accumulation in a concentration‐dependent manner, transient overexpression (OE) and virus‐induced gene silencing (VIGS) assays were performed in ‘Suli’ pear fruits at 35 DAFB, followed by treatment with 4% (high‐energy) or 1% (low‐energy) glucose for 7 days. Under 4% glucose, phloroglucinol‐HCl staining revealed weaker lignin deposition at *PbSnRK1*‐OE sites compared with empty vector (EV) controls, whereas *PbSnRK1*‐VIGS sites showed enhanced staining (Figure 1f). Quantification analysis confirmed that *PbSnRK1* overexpression reduced lignin and stone cell contents, while silencing promoted their accumulation (Figure 1g,h). Under 1% glucose, the opposite effects were observed (Figure 1i–k). Notably, in the absence of exogenous glucose, no significant differences were detected among any of the experimental lines (Figure S4a). RT‐qPCR further confirmed that *PbSnRK1* expression significantly modified the transcript levels of lignin biosynthetic genes under glucose treatment (Figure S4b–e). These results demonstrate that *PbSnRK1* bidirectionally regulates lignin biosynthesis in a strictly glucose‐dependent manner.

Previous studies have demonstrated that phosphorylation of the threonine residue (Thr‐175) in the T‐loop of the SnRK1 catalytic subunit is crucial for its kinase activity, and T175 mutations result in complete inactivation in Arabidopsis (Broeckx et al. 2016). To further confirm this regulatory role and assess its dependence on kinase activity, stable transgenic pear calli overexpressing *PbSnRK1* (*PbSnRK1*‐OE) and a kinase‐dead mutant *PbSnRK1* ^*T175A*^‐OE were generated (Figure S5a). Consistent with the transient fruit assay results, no significant difference in lignin content was detected among EV‐OE, *PbSnRK1*‐OE and *PbSnRK1* ^*T175A*^‐OE calli under glucose‐free conditions (Figure S5b). However, under 4% glucose treatment, *PbSnRK1*‐OE calli showed weaker lignin staining and reduced lignin content compared with EV‐OE calli, whereas no significant difference was detected between *PbSnRK1* ^*T175A*^‐OE calli and the EV‐OE (Figure 1l,m; Figure S5c). Conversely, under 1% glucose treatment, *PbSnRK1*‐OE calli displayed stronger lignin staining and elevated lignin content relative to EV, while *PbSnRK1* ^*T175A*^‐OE calli showed no significant changes (Figure 1n,o; Figure S5d). These results indicate that *PbSnRK1* mediates glucose‐dependent bidirectional regulation of lignin accumulation, and its kinase activity is indispensable for both negative regulation under high glucose and positive regulation under low glucose conditions.

Additionally, *PbSnRK1* was heterologously overexpressed in *Arabidopsis thaliana* to verify its function. Under 4% glucose treatment, *PbSnRK1*‐OE lines showed increased root elongation and plant height, accompanied by reduced lignin content and downregulated expression of secondary cell wall (SCW) biosynthetic genes (Figure 1p; Figure S6a–d). In contrast, under 1% glucose treatment, opposite phenotypes were observed (Figure 1q; Figure S6e–g). No significant differences in lignin content were detected between WT and transgenic lines under glucose‐free conditions (Figure S6h). Toluidine blue O and safranin O‐fast green staining further confirmed that *PbSnRK1*‐OE lines exhibited thinner SCWs in interfascicular fibres and xylem under 4% glucose, but thicker SCWs under 1% glucose (Figure 1r–u; Figure S6i,j). Collectively, these results demonstrate that *PbSnRK1* exerts conserved, glucose‐dependent bidirectional regulation of lignin biosynthesis and SCW thickness across plant species. Thus, glucose concentration mediates the biphasic regulation of lignin biosynthesis via the PbSnRK1 kinase during pear fruit development.

### 2.3 PbSnRK1 Differentially Regulates PbTCP13 to Modulate Lignin Biosynthesis in a Glucose‐Dependent Manner

To elucidate the molecular mechanism by which *PbSnRK1* transduces glucose signals to regulate lignin biosynthesis, a yeast two‐hybrid (Y2H) screen was performed using the PbSnRK1 coding sequence as bait against a pear fruit cDNA library. This screening identified nine candidate interactors (Table S4), among which *PbTCP13* (Pbr026562.3), a member of the Class I TCP subfamily (Figure S7a), was selected for further analysis. Phylogenetic analysis revealed that PbTCP13 is most closely related to Arabidopsis AtTCP8 and apple MdTCP21 (Figure S7b), which have been reported to regulate cell proliferation and shoot branching, with no known involvement in lignin regulation. Y2H, Luciferase complementation imaging (LCI), and bimolecular fluorescence complementation (BiFC) assays validated this interaction in vivo (Figure 2a–c). Pull‐down assay further confirmed this finding in vitro (Figure 2d). Additionally, *PbTCP13* expression was significantly decreased under 4% glucose treatment (Figure S7c), and *PbTCP13* localized to the nucleus (Figure S7d), consistent with its function as a nuclear TF that modulates downstream lignin‐related pathways. Taken together, these results confirm that PbSnRK1 interacts with PbTCP13 both in vitro and in vivo, and that the two proteins co‐localize in the nucleus.

**FIGURE 2:**
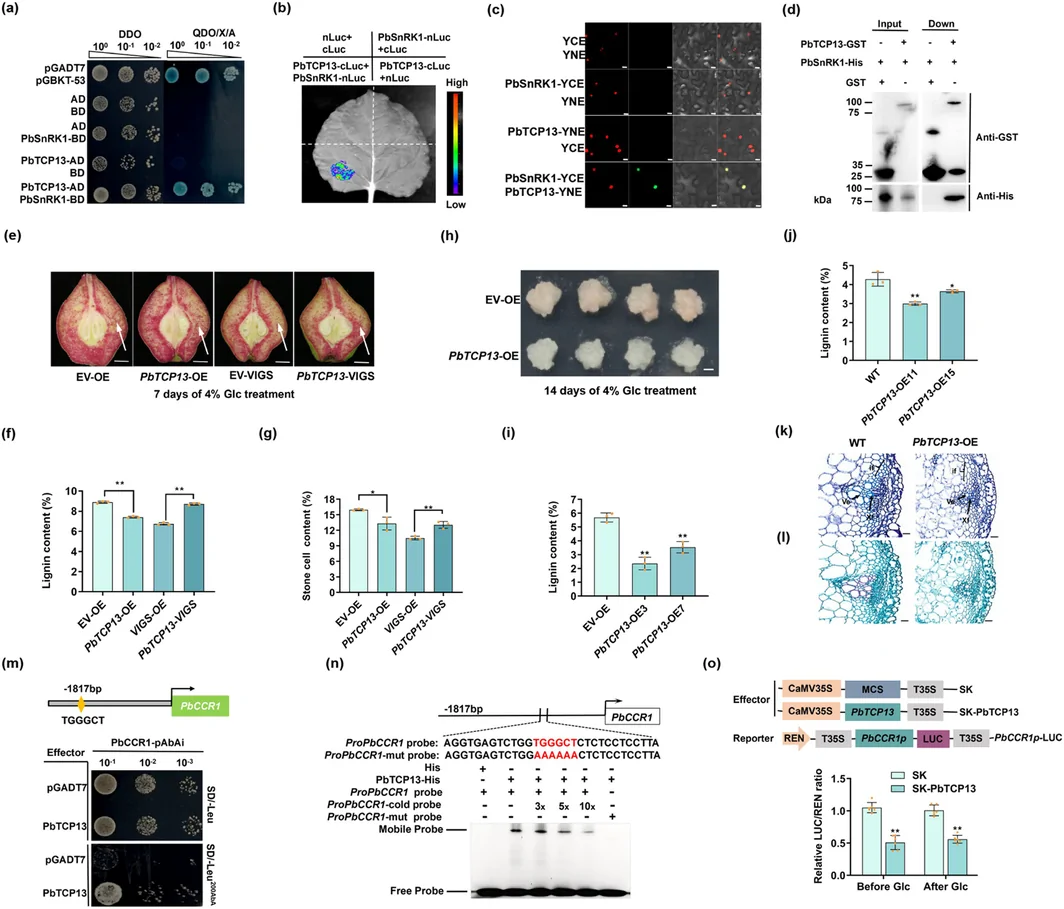
*PbSnRK1* differentially regulates *PbTCP13* to modulate lignin biosynthesis in a glucose‐dependent manner. Physical interaction between PbSnRK1 and PbTCP13 was verified by (a) Y2H, (b) LCI, (c) BiFC and (d) GST pull‐down assays. (e) Phloroglucinol‐HCl staining of EV‐OE, *PbTCP13*‐OE, EV‐VIGS and *PbTCP13*‐VIGS transgenic pear fruits following 4% glucose treatment (scale bar, 0.5 cm). (f) Lignin content and (g) stone cell content in the above transgenic pear fruits. (h) Phloroglucinol‐HCl staining of EV‐OE and *PbTCP13*‐OE transgenic pear calli following 4% glucose treatment (scale bar, 0.5 cm) and (i) lignin content. (j) Lignin content of *PbTCP13‐*overexpressing Arabidopsis under 4% glucose treatment. (k) Toluidine blue O and (l) safranin O‐fast green staining of cross‐sections from inflorescence stems of WT and *PbTCP13*‐OE Arabidopsis under 4% glucose treatment (scale bar, 25 μm). ve, vessel; xf, xylary fibre; if, interfascicular fibre. (m) A Y1H assay indicated that PbTCP13 can bind to the *PbCCR1* promoter. (n) EMSA confirmed that PbTCP13 binds to the TGGGCT motif in the *PbCCR1* promoter. (o) Dual‐luciferase reporter assays in *N. benthamiana* leaves showed that *PbTCP13* represses the transcriptional activity of the *PbCCR1* promoter. Data are presented as means ± SE (*n* = 3 for f, g, i, j; *n* = 6 for o). Statistical significance was determined by two‐tailed Student's *t*‐test (*p < 0.05; **p < 0.01).

To characterize the function of *PbTCP13* in glucose‐responsive lignin deposition, transient overexpression and VIGS assays were performed in ‘Suli’ pear fruit at 35 DAFB. After 7 days of 4% glucose treatment, *PbTCP13*‐OE sites exhibited attenuated lignin staining, whereas *PbTCP13*‐VIGS regions displayed intensified staining compared with EV‐OE (Figure 2e). Accordingly, *PbTCP13*‐OE fruit accumulated lower lignin and stone cell contents, while *PbTCP13*‐VIGS fruit exhibited elevated levels (Figure 2f,g). RT‐qPCR analysis confirmed that altered *PbTCP13* expression significantly affected the transcription of lignin biosynthetic genes (Figure S8a,b). Under normal growth conditions (without exogenous glucose treatment), no significant differences in lignin accumulation were observed between transgenic and control lines (Figure S8c). Consistent with these findings, *PbTCP13*‐OE transgenic pear calli revealed that the control pear calli turned red and accumulated large amounts of lignin after a 14‐day 4% glucose treatment, whereas the *PbTCP13*‐OE pear calli remained pale with relatively little accumulated lignin (Figure 2h,i). RT‐qPCR analysis further confirmed that altered *PbTCP13* expression resulted in corresponding changes in lignin biosynthetic gene expression (Figure S8d). No significant difference in lignin content was detected between the two lines under glucose‐free conditions (Figure S8e). Heterologous expression of *PbTCP13* in *Arabidopsis thaliana* further validated this high glucose‐specific regulatory function (Figure S8f). When grown on 4% glucose‐containing medium, two independent transgenic lines displayed increased root elongation and plant height, accompanied by reduced lignin content and downregulated expression of SCW biosynthetic genes (Figure 2j; Figure S8f–i). Consistent with the pear results, lignin accumulation was comparable between WT and transgenic lines under glucose‐free conditions (Figure S8j). Histological staining confirmed that *PbTCP13* overexpression reduced lignification in interfascicular fibres and xylem vessels, corresponding to thinner SCWs in transgenic plants (Figure 2k,l; Figure S8k).

To elucidate the molecular mechanism underlying *PbTCP13*‐mediated lignin repression, the effect of *PbTCP13* on the expression of key lignin biosynthetic genes was examined. *PbCCR1* expression was significantly downregulated by *PbTCP13* overexpression and upregulated following *PbTCP13* silencing in pear fruits and pear calli. Yeast one‐hybrid (Y1H) and electrophoretic mobility shift assay (EMSA) assays verified that PbTCP13 specifically binds to the TGGGCT motif in the *PbCCR1* promoter (Figure 2m,n). Dual‐luciferase (Dual‐LUC) assays in *Nicotiana benthamiana* further demonstrated that *PbTCP13* significantly represses *PbCCR1* promoter activity, and this inhibitory effect was maintained under 4% glucose treatment (Figure 2o). Collectively, these results demonstrate that *PbTCP13* functions as a transcriptional repressor that binds to the *PbCCR1* promoter and inhibits lignin biosynthesis specifically under high glucose conditions.

### 2.4 PbSnRK1 Phosphorylates and Destabilizes PbTCP13 in Response to Glucose

To explore the functional impact of the PbSnRK1‐PbTCP13 interaction, we assessed whether PbSnRK1 can phosphorylate PbTCP13 via in vitro kinase assays. Due to the insufficient purity of the full‐length PbSnRK1 for in vitro kinase assays, we purified a truncated variant containing the conserved S_TKc kinase domain and UBA domain, fused to a maltose‐binding protein (MBP) tag. Incubation of purified MBP‐PbSnRK1 with His‐tagged PbTCP13 resulted in a distinct phosphorylation signal for His‐PbTCP13, whereas no signal was detected in the absence of kinase (Figure 3a), demonstrating that PbSnRK1 directly phosphorylates PbTCP13 in vitro. To identify the phosphorylation site(s), we performed LC–MS/MS analysis of the in vitro phosphorylated protein, which identified Ser265 as a candidate residue (Figure 3b). Notably, this residue is conserved as a serine or threonine among TCP13 homologues from diverse plant species (Figure S7e), both of which are compatible with the phospho‐acceptor position of the SnRK1 recognition motif, supporting its functional importance. To determine whether this site represents the primary target of PbSnRK1‐mediated phosphorylation, we generated a mutant PbTCP13 (S265A) by substituting Ser265 with alanine. In vitro kinase assays demonstrated that this substitution almost abolished PbSnRK1‐mediated phosphorylation of PbTCP13 (Figure 3c), confirming Ser265 as a primary phosphorylation site targeted by PbSnRK1.

**FIGURE 3:**
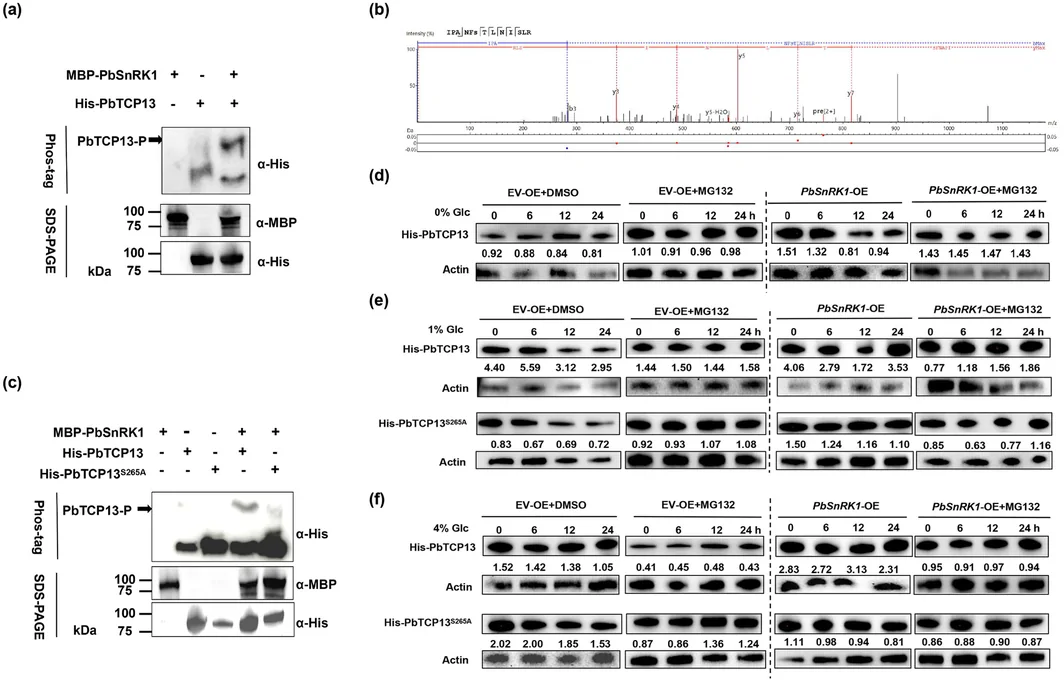
PbSnRK1 phosphorylates and destabilizes PbTCP13 in response to glucose. (a) In vitro kinase assay showed that PbSnRK1 phosphorylated PbTCP13. Phosphorylated PbTCP13 (PbTCP13‐P) was detected by Phos‐tag gel assay (top panel). The levels of PbTCP13 (anti‐His) and PbSnRK1 (anti‐MBP) proteins were detected by SDS‐PAGE gels. (b) LC‐tandem mass spectrometry (MS/MS) analysis identified the phosphorylation sites of PbTCP13 by PbSnRK1 in vitro. (c) Phos‐tag gel assay verified that PbSnRK1 mediates the phosphorylation of PbTCP13 at Ser265. The phosphorylation status of PbTCP13 and PbTCP13^S265A^ was examined in a Phos‐tag gel assay (top panel). The levels of PbTCP13/PbTCP13^S265A^ (anti‐His) and PbSnRK1 (anti‐MBP) proteins were shown on the SDS‐PAGE gels. (d) Cell‐free degradation assay showed the stability of PbTCP13 in protein extracts from transgenic pear calli. (e, f) Cell‐free degradation assays showed the stability of wild‐type PbTCP13 and PbTCP13^S265A^ under (e) 1% and (f) 4% glucose treatment, respectively.

As protein phosphorylation commonly regulates protein stability, we used cell‐free degradation assays to determine whether PbSnRK1‐mediated phosphorylation affects PbTCP13 degradation. Wild‐type PbTCP13 protein was degraded significantly more rapidly in extracts from *PbSnRK1*‐OE pear calli than in those from EV‐OE (Figure 3d). This degradation was effectively blocked by the 26S proteasome inhibitor MG132, indicating that PbTCP13 is degraded via the ubiquitin‐proteasome pathway. Notably, the degradation rate of PbTCP13 was dependent on the glucose concentration. Under 1% glucose conditions, the PbTCP13^S265A^ mutant exhibited a markedly slower degradation rate than wild‐type PbTCP13 (Figure 3e). In contrast, no significant difference in degradation rate was observed between the S265A mutant and the wild‐type PbTCP13 under 4% glucose conditions (Figure 3f). Collectively, these results indicate that PbSnRK1 phosphorylates PbTCP13 at Ser265 to accelerate its proteasomal degradation, and this regulatory effect is pronounced under low‐glucose conditions where PbSnRK1 is active and attenuated under high‐glucose conditions where PbSnRK1 is inhibited.

### 2.5 Identification of PbMYB11 as a PbTCP13‐Interacting Transcriptional Activator

Given that *PbSnRK1*‐OE promotes lignin accumulation under 1% glucose, we hypothesized that SnRK1 signalling activates a transcriptional activator of lignin biosynthesis under low‐glucose conditions. To identify such factors, RNA sequencing (RNA‐seq) was performed on *PbSnRK1*‐OE and EV‐OE pear calli treated with 1% or 4% glucose. Weighted gene co‐expression network analysis (WGCNA) identified 10 co‐expression modules, among which the ‘plum3’ module exhibited the strongest correlation with lignin accumulation traits (Figure S9). This module included six MYB TFs (Table S1), among which *PbMYB11* (pycom02g14500) showed the highest expression in *PbSnRK1*‐OE calli under 1% glucose (Figure S10a). Therefore, *PbMYB11* was selected for further functional characterization. *PbMYB11* expression level was significantly upregulated under 1% glucose treatment (Figure 4a). Phylogenetic analysis revealed that PbMYB11 clusters in a distinct clade with *Arabidopsis thaliana* AtMYB73 and *Vitis vinifera* VvMYB44 (Figure S10b), which have been reported to function as a salt stress‐responsive repressor and an ABA and sugar‐related metabolic activator, respectively (Kim et al. 2013; Liu et al. 2026). This clade is evolutionarily separated from canonical lignin transcriptional activators such as AtMYB46. PbMYB11 possessed conserved R2 and R3 MYB repeats at the N‐terminus (Figure S10c) and localized exclusively to the nucleus (Figure 4b), consistent with its predicted function as a DNA‐binding TF.

**FIGURE 4:**
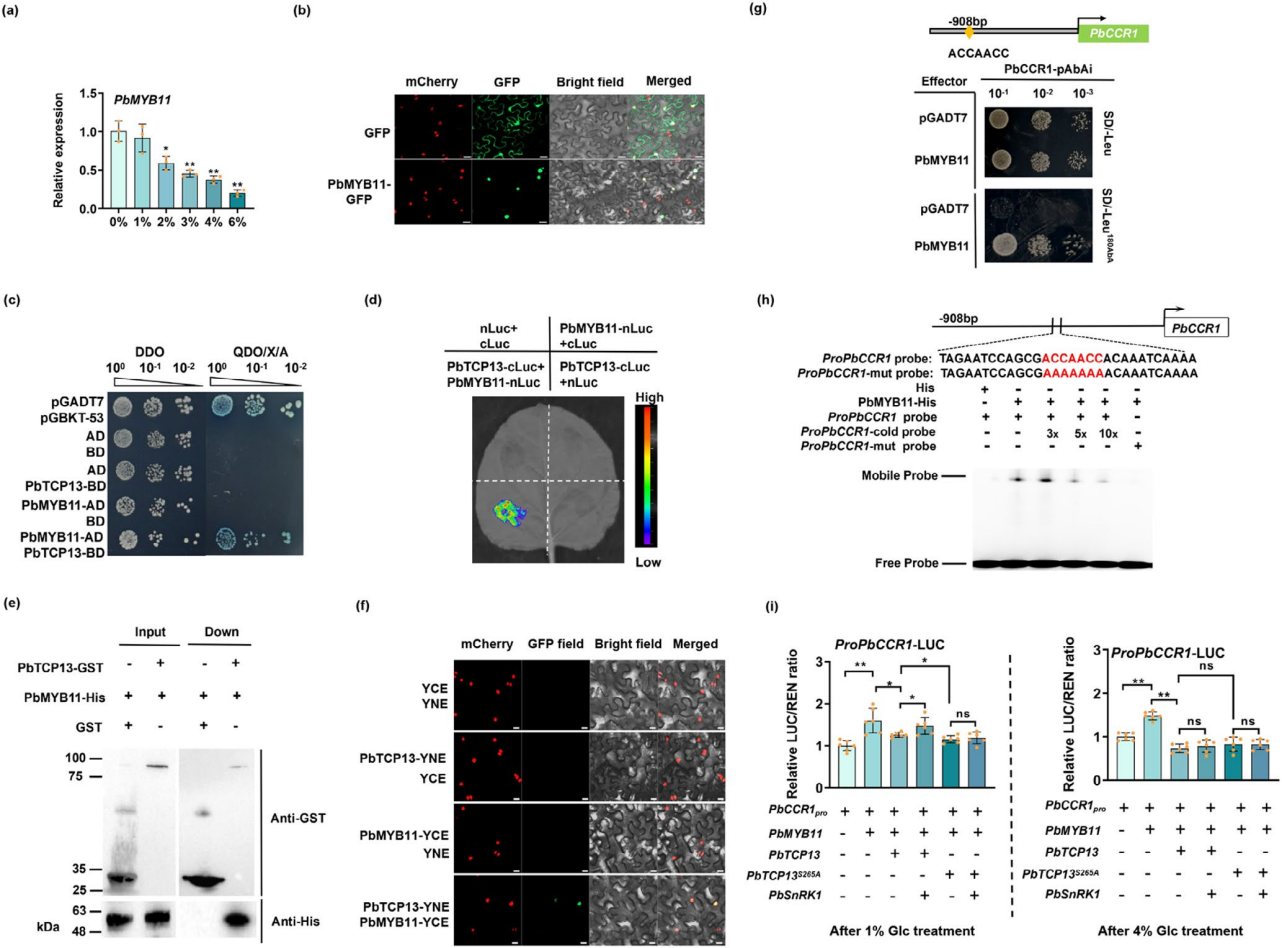
PbSnRK1 phosphorylates PbTCP13 at Ser265 to regulate its interaction with PbMYB11 and *PbCCR1* transcription under varying glucose conditions. (a) Expression levels of *PbMYB11* in pear calli under 0%, 1%, 2%, 3%, 4%, or 6% (w/v) glucose treatment. (b) Subcellular localization showed that PbMYB11 was localized in the nucleus of *N. benthamiana* leaf cells (scale bar, 20 μm). Physical interaction between PbMYB11 and PbTCP13 was verified by (c) Y2H, (d) LCI, (e) GST pull‐down and (f) BiFC assays. (g) Y1H assay confirmed that PbMYB11 binds to the AC elements within the *PbCCR1* promoter region. (h) EMSA confirmed the specific binding ability of PbMYB11 to the AC elements in the *PbCCR1* promoter. (i) Dual‐luciferase reporter assays in *N. benthamiana* leaves verified that S265 in PbTCP13 affected its transcriptional abilities under 1% and 4% glucose conditions. Data are presented as means ± SE (*n* = 3 for a; *n* = 6 for i). Statistical significance was determined by two‐tailed Student's *t*‐test (n.s., no significance, *p* > 0.05; *p < 0.05; **p < 0.01).

To assess how *PbSnRK1* and *PbTCP13* affect *PbMYB11*, transcript levels were examined in stable transgenic calli. Under 4% glucose treatment, *PbMYB11* expression was 2.3‐fold higher in *PbSnRK1*‐OE calli than in EV‐OE calli, whereas *PbTCP13*‐OE had no significant effect on *PbMYB11* transcript levels (Figure S10d). These results suggest that *PbMYB11* transcription is responsive to SnRK1 signalling but is not directly repressed by *PbTCP13* at the transcript level. Y2H assays verified a direct physical interaction between PbTCP13 and PbMYB11 (Figure 4c). The interaction was further confirmed by LCI assays, GST pull‐down, and BiFC, which revealed that the two proteins interacted both in vivo and in vitro (Figure 4d–f). Taken together, these results demonstrate that PbTCP13 physically interacts with PbMYB11 in the nucleus.

### 2.6 Phosphorylation of PbTCP13 at Ser265 Regulates Its Interaction With PbMYB11 and *PbCCR1* Promoter Activity

To explore the role of PbSnRK1‐mediated phosphorylation of PbTCP13 at Ser265, we assessed its impact on subcellular localization, protein–protein interaction, DNA‐binding activity, and transcriptional regulatory activity. Subcellular localization assays showed that the GFP signals of 35S: *PbTCP13* ^*S265A*^‐GFP exhibited an identical nuclear localization pattern to those of wild‐type 35S: *PbTCP13*‐GFP (Figure S11a), demonstrating that phosphorylation at Ser265 has no significant impact on PbTCP13 subcellular localization. Subsequently, BiFC and LCI assays were performed to determine whether Ser265 phosphorylation affects the protein–protein interaction between PbTCP13 and PbMYB11. Both assays revealed that the S265A mutation partially attenuated the interaction intensity between PbTCP13 and PbMYB11, although the interaction was not completely abrogated (Figure S11b,c), indicating that Ser265 phosphorylation modulates the physical association of the two proteins.

To investigate the regulatory role of *PbMYB11* on *PbCCR1* transcription and whether this regulation is coordinated by PbSnRK1‐mediated phosphorylation of PbTCP13 at Ser265, Y1H and EMSA assays were performed. These assays demonstrated that PbMYB11 binds to AC elements in the *PbCCR1* promoter (Figure 4g,h). Dual‐luciferase assays in *Nicotiana benthamiana* leaves showed that *PbMYB11* significantly enhanced the *PbCCR1* promoter activity (Figure S11d), identifying *PbMYB11* as a transcriptional activator targeting the AC motifs of *PbCCR1*. As described above, *PbTCP13* binds the TGGGCT motif in the *PbCCR1* promoter to repress its transcription (Figure 2m–o). To clarify the glucose‐dependent regulatory role of the PbSnRK1‐PbTCP13 module, dual‐luciferase assays were performed under low‐glucose (1%) and high‐glucose (4%) conditions. The results revealed that co‐expression of *PbMYB11* increased *PbCCR1* promoter‐driven luciferase activity under both low and high glucose conditions. Meanwhile, co‐expression of wild‐type *PbTCP13* markedly suppressed the *PbMYB11*‐mediated transcriptional activation of the *PbCCR1* promoter at both glucose levels. Notably, in the absence of *PbSnRK1* co‐expression, *PbTCP13* ^*S265A*^ and wild‐type *PbTCP13* showed comparable repressive effects (Figure 4i). Under low glucose (1%) conditions, co‐expression of *PbSnRK1* partially relieved the repression mediated by wild‐type *PbTCP13*, but this relief was abolished when *PbTCP13* ^*S265A*^ was co‐expressed instead. Furthermore, co‐expression of *PbSnRK1* did not relieve the *PbTCP13*‐mediated repression under 4% glucose conditions (Figure 4i). Taken together, these results demonstrate that PbSnRK1‐mediated phosphorylation of PbTCP13 at Ser265 modulates *PbCCR1* transcription by regulating the functional interplay between repressive *PbTCP13* and activating *PbMYB11* in a glucose concentration‐dependent manner.

### 2.7 PbMYB11 Positively Regulates Lignin Biosynthesis Under Low‐Glucose Conditions

To characterize the function of *PbMYB11* in pear lignin biosynthesis, transient overexpression and VIGS assays were performed in ‘Suli’ pear fruit at 35 DAFB. Following 7 days of 1% glucose treatment, *PbMYB11*‐OE injection sites showed significantly stronger lignin staining than EV‐OE, whereas staining was markedly attenuated in *PbMYB11*‐VIGS regions (Figure 5a). Quantitative analysis confirmed that *PbMYB11* overexpression significantly increased lignin and stone cell contents, while *PbMYB11* silencing induced the opposite phenotype (Figure 5b,c). RT‐qPCR analysis further demonstrated that transcript levels of lignin biosynthetic genes, including that of the key target *PbCCR1*, changed correspondingly with *PbMYB11* expression levels (Figure S12a,b). Under 4% glucose or glucose‐free conditions, no significant differences in lignin accumulation were detected between *PbMYB11*‐OE/VIGS lines and EV controls (Figure S12c–h). Suggesting that *PbMYB11* functions as a positive regulator specific under low‐glucose conditions for lignin biosynthesis in pear fruit.

**FIGURE 5:**
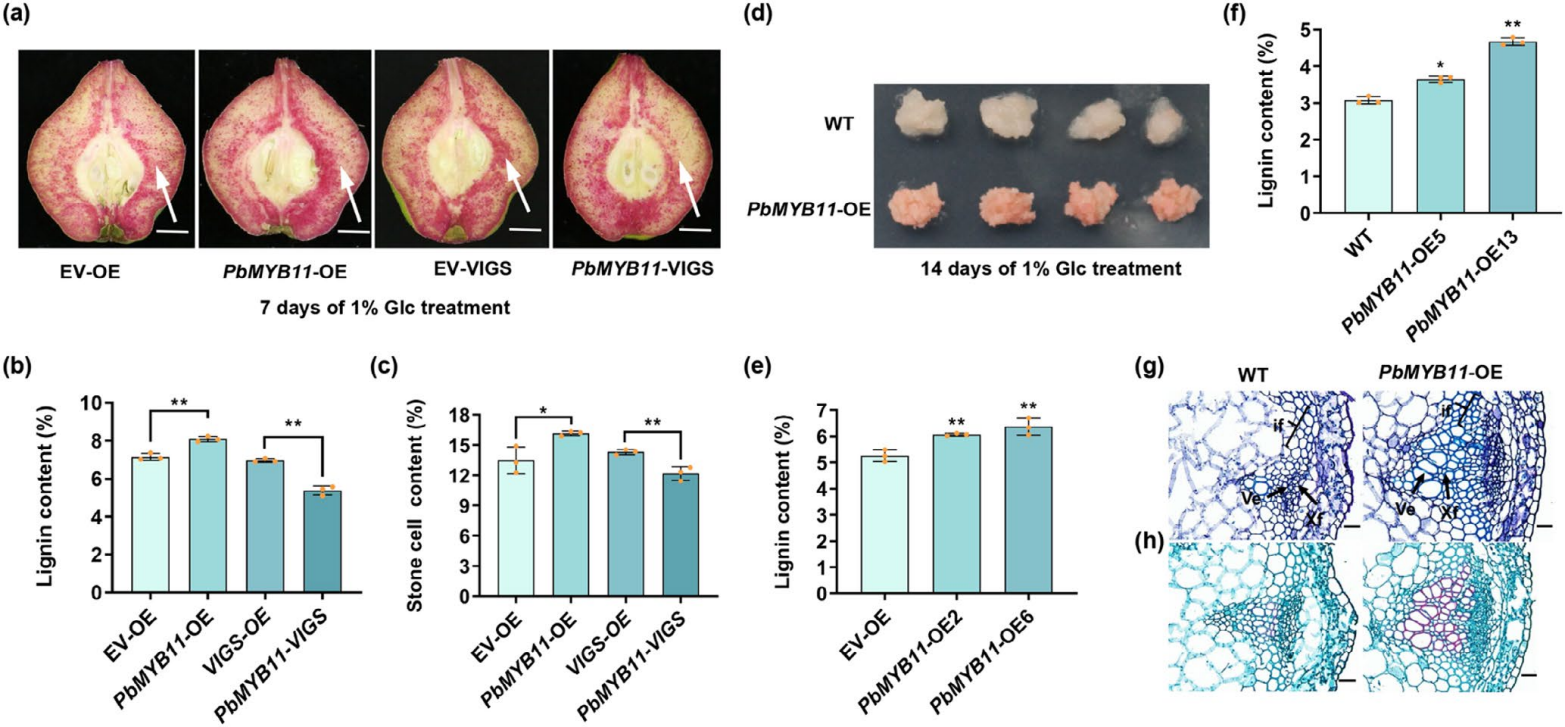
PbMYB11 positively regulates lignin biosynthesis under low glucose conditions. (a) Phloroglucinol‐HCl staining of EV‐OE, *PbMYB11*‐OE, EV‐VIGS and *PbMYB11*‐VIGS transgenic pear fruits following 1% glucose treatment for 7 days (scale bar, 0.5 cm). (b) Lignin content and (c) stone cell content in the above transgenic pear fruits. (d) Phloroglucinol‐HCl staining of EV‐OE and *PbMYB11*‐OE stable transgenic pear calli following 1% glucose treatment for 14 days (scale bar, 1 cm) and (e) lignin content. (f) Lignin content of WT and *PbMYB11*‐OE Arabidopsis under 1% glucose treatment. (g) Toluidine blue O and (h) safranin O‐fast green staining of cross‐sections from inflorescence stems of WT and *PbMYB11*‐OE Arabidopsis under 1% glucose treatment (scale bar, 25 μm). ve, vessel; xf, xylary fibre; if, interfascicular fibre. Data are presented as means ± SE (*n* = 3). Statistical significance was determined by two‐tailed Student's *t*‐test (*p < 0.05; **p < 0.01).

To further validate these findings, stable transgenic pear calli overexpressing *PbMYB11* were generated for further validation. Following 14 days of 1% glucose treatment, *PbMYB11*‐OE calli showed higher lignin content and significantly stronger lignin staining than EV‐OE calli (Figure 5d,e), accompanied by upregulated expression of *PbCCR1* and other lignin biosynthetic genes (Figure S13a). No significant differences in lignin accumulation were detected between *PbMYB11*‐OE and EV‐OE calli under 4% glucose or normal growth conditions (Figure S13b–e), further confirming the low‐glucose‐specific regulatory role of *PbMYB11*.

In addition, *PbMYB11* was heterologously expressed in *A. thaliana* (Figure S14a). Under 1% glucose, two independent *PbMYB11*‐OE lines exhibited significantly reduced root length and plant height, accompanied by elevated lignin content and upregulated expression of SCW biosynthetic genes (Figure 5f; Figure S14b–d). Lignin content was comparable between *PbMYB11*‐OE lines and WT under glucose‐free conditions (Figure S14e). Toluidine blue O and safranin O‐fast green staining revealed increased lignin deposition in interfascicular fibres and xylem of *PbMYB11*‐OE lines under 1% glucose, with thicker SCWs in vessel cells (Figure 5g,h). Collectively, these results demonstrate that *PbMYB11* functions as a low‐glucose‐specific positive regulator of lignin accumulation and secondary cell wall thickening.

### 2.8 The PbSnRK1‐PbTCP13‐PbMYB11 Module Mediates Glucose‐Responsive Lignin Biosynthesis in Pear

To elucidate the genetic hierarchy and functional dependency within the PbSnRK1‐PbTCP13‐PbMYB11 module in glucose‐modulated lignin regulation, genetic assays were performed using stable transgenic pear calli. Multiple calli lines were generated, including empty vector control (EV‐OE), *PbTCP13*‐OE, *PbTCP13*‐OE/*PbSnRK1*‐OE and *PbTCP13‐*OE/*PbMYB11*‐OE, and treated with 1% (low) or 4% (high) glucose. Under low glucose conditions, *PbSnRK1*‐OE calli displayed enhanced lignin staining and increased lignin content compared to EV‐OE, while *PbTCP13*‐OE calli exhibited the opposite phenotype. Notably, *PbSnRK1*‐OE/*PbTCP13*‐OE co‐expression lines restored lignin levels to those of EV controls. Furthermore, the inhibitory effect of *PbTCP13* overexpression on lignin accumulation was largely reversed by co‐overexpression of *PbMYB11*, consistent with both staining and quantitative results (Figure 6a,b). Under high glucose conditions, *PbSnRK1*‐OE/*PbTCP13*‐OE lines exhibited lignin content comparable to *PbSnRK1*‐OE single lines, both of which were significantly lower than EV controls. Conversely, lignin content in *PbTCP13*‐OE/*PbMYB11*‐OE lines was comparable to EV controls and ranked between the levels of *PbTCP13*‐OE and *PbMYB11*‐OE single lines (Figure S15a). RT‐qPCR analysis further revealed that, under 1% glucose, lignin biosynthetic structural genes were significantly upregulated in *PbSnRK1*‐OE calli and downregulated in *PbTCP13*‐OE calli, and returned to control levels in both co‐overexpression lines, fully consistent with the lignin phenotypes (Figure S15b).

**FIGURE 6:**
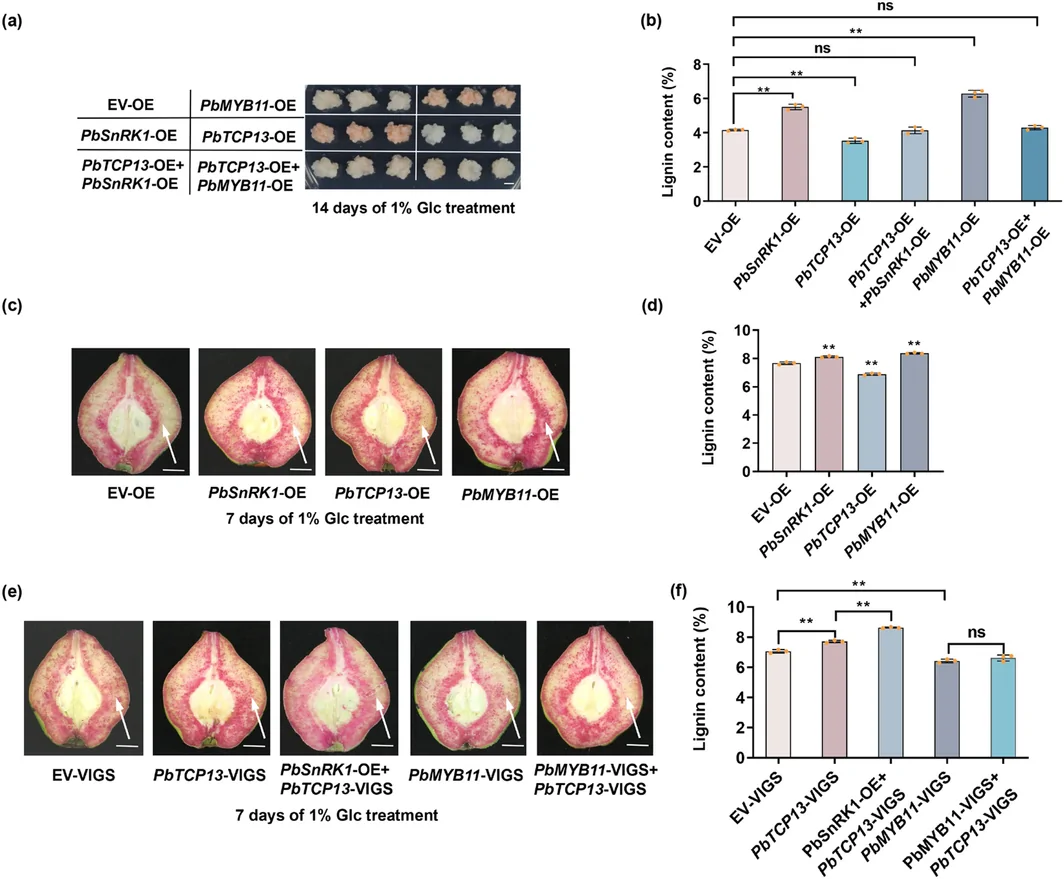
The PbSnRK1‐PbTCP13‐PbMYB11 module mediates glucose‐responsive lignin biosynthesis in pear. (a) Phloroglucinol‐HCl staining of EV‐OE, *PbTCP13*‐OE, *PbSnRK1*‐OE, *PbMYB11*‐OE, *PbTCP13*‐OE/*PbSnRK1*‐OE and *PbTCP13*‐OE/*PbMYB11*‐OE transgenic pear calli following 1% glucose treatment (scale bar, 1 cm) and (b) lignin content. (c) Phloroglucinol‐HCl staining of EV‐OE, *PbSnRK1*‐OE, *PbTCP13*‐OE and *PbMYB11*‐OE transgenic pear fruits under 1% glucose treatment (scale bar, 0.5 cm) and (d) lignin content. (e) Phloroglucinol‐HCl staining of EV‐VIGS, *PbTCP13*‐VIGS, *PbSnRK1*‐OE/*PbTCP13*‐VIGS, *PbMYB11*‐VIGS and *PbMYB11*‐VIGS/*PbTCP13*‐VIGS transgenic pear fruits under 1% glucose treatment (scale bar, 0.5 cm) and (f) lignin content. Data are presented as means ± SE (*n* = 3). Statistical significance was determined by two‐tailed Student's *t*‐test (n.s., no significance, *p* > 0.05; **p < 0.01).

Transient co‐transformation assays were subsequently performed in pear fruit to validate these genetic relationships. Consistent with the calli results, *PbSnRK1* overexpression significantly enhanced lignin staining and increased lignin content in pear fruit under low glucose conditions (Figure 6c,d), and this promoting effect was further strengthened by simultaneous silencing of *PbTCP13* (Figure 6e). Conversely, silencing *PbMYB11* alone markedly reduced lignin deposition, and this reduction could not be rescued by co‐silencing *PbTCP13* (Figure 6e,f). The expression patterns of key lignin biosynthetic genes were consistent with the phenotypic changes (Figure S15c,d). Consistent with the calli results, no significant differences in lignin content were detected among these transgenic lines under 4% glucose treatment (Figure S15e,f). Taken together, these genetic results demonstrate that *PbTCP13* acts genetically downstream of *PbSnRK1* and upstream of *PbMYB11*, and that this three‐component cascade mediates glucose concentration‐dependent regulation of lignin biosynthesis in pear.

## 3 Discussion

Since lignin deposition is an irreversible and energetically costly process, plants have evolved precise regulatory strategies to coordinate secondary cell wall biosynthesis with dynamic cellular carbon and energy availability. Beyond serving as a primary energy source and a carbon precursor for monolignol synthesis, glucose acts as a core signalling molecule that governs plant developmental and metabolic reprogramming (Rolland et al. 2006; Lastdrager et al. 2014). Previous studies in Arabidopsis stems have revealed that sugar availability modulates the diurnal rhythm of lignin biosynthetic gene expression, indicating that glucose regulates lignification beyond basal nutritional supply (Rogers et al. 2005). Consistent with recent findings in grapevine stems documenting a positive correlation between sugar availability and lignin accumulation (Wang, Huang, et al. 2024; Wang, Wang, et al. 2024), our results confirm that glucose promotes lignin biosynthesis in pear calli in a dose‐dependent manner. Moreover, equiosmotic mannitol control assays excluded nonspecific osmotic stress effects, verifying that the lignin‐promoting phenotype is specifically triggered by glucose signalling rather than general osmotic responses (Figure S1). Furthermore, we observed a stable inverse association between glucose accumulation and lignin deposition both across a natural population of 14 pear cultivars with divergent stone cell traits and throughout the entire developmental cycle of ‘Suli’ pear (Figure 1b–d; Figure S3). Stratified analysis further showed that this negative association was more pronounced in high‐stone cell cultivars with a wider dynamic range of lignin accumulation (Figure S3). These in vivo findings indicate that endogenous glucose acts as a conserved developmental cue that aligns the temporal progression of stone cell lignification with cellular carbon status in pear fruit across diverse genotypic backgrounds. Consistent with this conclusion, a previous study in pear also noted that endogenous glucose accumulation coincides temporally with active stone cell differentiation at early fruit development (Gong et al. 2023), further supporting the role of glucose as an endogenous regulator of stone cell lignification. Collectively, these findings establish glucose as a concentration‐dependent regulator of stone cell lignification in pear, linking cellular carbon status to secondary cell wall deposition in fleshy fruits.

The dose‐dependent regulation of lignin biosynthesis by glucose implies the existence of a core molecular hub that translates cellular carbon availability into secondary cell wall deposition outputs. As a conserved eukaryotic energy sensor, SnRK1 functions as a metabolic fuel gauge that is activated by energy deficiency and repressed by high sugar abundance (Baena‐González et al. 2007; Emanuelle et al. 2015; Wang et al. 2019). Here, we found that the pear *PbSnRK1* transcript abundance is dynamically modulated by glucose in a dose‐responsive manner in pear calli (Figure S2a). Consistent with this in vitro regulatory pattern, in vivo developmental profiling of ‘Suli’ pear further showed that *PbSnRK1* transcript levels were negatively correlated with endogenous glucose content, with its expression peak coinciding exactly with the rapid lignin accumulation phase at early fruit development (Figure 1e), supporting its role as an endogenous glucose signal transducer in pear fruit. Functional assays further revealed a dual regulatory role for *PbSnRK1* in lignin deposition: *PbSnRK1* promoted lignin accumulation under low glucose (1%) conditions, whereas it suppressed lignification under high glucose (4%) conditions (Figure 1). This bidirectional regulation was strictly kinase activity‐dependent, as the kinase‐dead T175A mutation completely abolished the regulatory function of *PbSnRK1* (Figure 1l–o). Consistently, SnRK1‐specific inhibitor T6P treatment further confirmed that glucose‐triggered lignin biosynthesis is partially dependent on PbSnRK1 kinase activity (Figure S2b–k). These results position PbSnRK1 as a central molecular hub coupling glucose signalling to lignin metabolism in pear fruit.

SnRK1 modulates plant metabolism primarily by phosphorylating downstream TFs to alter their stability and activity (Baena‐González et al. 2007; Mair et al. 2015; Hulsmans et al. 2016; Li and Liu 2021). Accumulating evidence indicates that SnRK1 regulates lignin biosynthesis through substrate‐specific mechanisms with divergent functional outputs: in tree peony and cassava, SnRK1 phosphorylates WRKY or HB TFs to enhance protein stability or activity, thereby positively promoting lignin biosynthesis (Yan et al. 2024; Luan et al. 2025); whereas in Arabidopsis, SnRK1 negatively regulates phenylpropanoid biosynthesis by triggering the proteasomal degradation of PAL, the rate‐limiting enzyme of the pathway (Wang et al. 2021). These divergent findings reflect the complexity of SnRK1‐mediated lignin regulation, yet the direct phosphorylation targets of SnRK1 that mediate stone cell lignification in perennial fruit trees such as pear remain largely uncharacterized. In this study, we identified *PbTCP13*, a Class I TCP TF, as a direct phosphorylation substrate of PbSnRK1. In vitro kinase assays demonstrated that recombinant PbSnRK1 directly phosphorylates PbTCP13 at the Ser265 residue, and mutation of this site to alanine (S265A) nearly abolished PbSnRK1‐mediated phosphorylation (Figure 3). This phosphorylation site matches the conserved SnRK1 consensus phosphorylation motif (Van Leene et al. 2022). It should be noted that these in vitro assays were performed using a truncated PbSnRK1 comprising only the S_TKc and UBA domains, owing to the insufficient purity of the full‐length protein for in vitro kinase assays. Although such truncated fragments have been shown to retain SnRK1 catalytic activity (Emanuelle et al. 2018; Zhi et al. 2026), we cannot fully exclude the possibility that the absence of the C‐terminal regulatory region affects the regulatory behaviour of PbSnRK1 in vivo. Cell‐free degradation assays further revealed that Ser265 phosphorylation is required for SnRK1‐triggered PbTCP13 turnover under low‐glucose conditions (Figure 3d–f). Consistent with analogous KIN10‐mediated TF degradation mechanisms in Arabidopsis (Zhai et al. 2017), our study reveals a conserved phosphorylation‐dependent regulatory mode in pear, wherein PbSnRK1 destabilizes PbTCP13 to relieve lignin repression under carbon‐limited conditions.

TCP TFs function broadly in plant development, with subfamily‐specific roles in secondary cell wall formation. Class II members predominantly promote lignin deposition: in Arabidopsis, TCP4 activates VND7 to trigger xylem vessel differentiation (Sun et al. 2017), and in pear, *PbTCP14* and *PbTCP15* are positively correlated with lignin accumulation and stone cell content (Zhao et al. 2021). Class I TCPs have also been implicated in secondary cell wall formation. For instance, *GhTCP14* and *GhTCP15* coordinate the transition to secondary cell wall synthesis in cotton fibre (Qiao et al. 2025). Our study identifies nucleus‐localized *PbTCP13* as a novel negative regulator of lignin biosynthesis in pear fruit (Figure 2e–l, Figure S8). Mechanistically, PbTCP13 binds the TGGGCT motif in the *PbCCR1* promoter to repress its transcription (Figure 2m–o). This finding expands the functional diversity of Class I TCPs and demonstrates antagonistic TCP subfamily activity that fine‐tunes lignin carbon allocation during fruit development. R2R3‐MYB TFs are core regulators of lignin biosynthesis, targeting AC‐rich cis‐elements in structural gene promoters to activate transcription (Zhong et al. 2008). Several pear MYBs have been reported to promote stone cell lignification (Xue et al. 2019, 2023; Gong et al. 2024). In this study, we identified *PbMYB11* as a positive regulator of lignin biosynthesis. Phylogenetic analysis clustered PbMYB11 with stress and metabolism‐related AtMYB73 and VvMYB44 (Figure S10b). Although this clade is separated from canonical secondary wall MYB activators, lignification is itself a conserved stress‐adaptive response (Moura et al. 2010), and stress‐responsive R2R3‐MYBs can directly activate lignin biosynthetic genes, as exemplified by *AtMYB15* in defence‐induced lignification in Arabidopsis (Chezem et al. 2017) and by *PcMYB44* in pathogen‐challenged pear (Yang et al. 2023). *PbMYB11* may thus represent a lineage‐specific co‐option of a stress‐ and sugar metabolism‐related MYB into the fruit‐specific programme of stone cell lignification. Consistent with this view, we demonstrated that PbMYB11 binds to AC elements in the *PbCCR1* promoter (Figure 4g,h). Functional validation through overexpression and silencing in pear fruit calli, together with heterologous expression in Arabidopsis, confirmed the conserved role of *PbMYB11* in promoting lignification and secondary wall thickening specifically under low‐glucose conditions (Figure 5). This context‐dependent function highlights the evolutionary plasticity of the phenylpropanoid pathway in Rosaceae, with specialized MYB members mediating carbon partitioning between competing metabolic branches.

The physical interaction between TCP and R2R3‐MYB TFs represents a conserved regulatory module in plant secondary metabolism. Previous studies in Arabidopsis showed that TCP3 directly interacts with multiple R2R3‐MYB proteins and enhances their transcriptional activation capacity to positively regulate flavonoid biosynthesis (Li and Zachgo 2013). In pear, PbTCP13 physically interacted with PbMYB11 both in vitro and in vivo, and BiFC assays further confirmed their interaction and co‐localization in the nucleus (Figure 4c–f). Dual‐luciferase assays showed that wild‐type *PbTCP13* represses *PbMYB11*‐mediated *PbCCR1* activation. Under low‐glucose conditions, PbSnRK1 partially relieved the repression mediated by wild‐type PbTCP13, but this relief was abolished when PbTCP13^S265A^ was co‐expressed, consistent with the requirement of Ser265 phosphorylation for PbTCP13 turnover and functional modulation (Figure 4i). Genetic epistasis assays further validated the hierarchical relationship of this regulatory module: *PbSnRK1* functions upstream of *PbTCP13*, while *PbMYB11* acts as the essential downstream effector of *PbTCP13* (Figure 6, Figure S15).

In summary, our study reveals a glucose concentration‐dependent mechanism that bidirectionally regulates stone cell lignification in pear. During early fruit development under low‐glucose conditions, activated PbSnRK1 phosphorylates PbTCP13 at Ser265 and triggers its degradation, thereby derepressing PbMYB11 to activate lignin biosynthetic gene expression and promote stone cell formation. With increasing glucose content during the mid‐to‐late stages of pear fruit development, suppressed PbSnRK1 activity stabilizes PbTCP13 protein, which restricts excessive lignification through both direct transcriptional repression of lignin biosynthetic structural genes and physical interaction with PbMYB11 to inhibit its function (Figure 7). This phosphorylation‐dependent molecular switch precisely adapts lignin biosynthesis to dynamic cellular carbon availability. Collectively, our findings establish the PbSnRK1‐PbTCP13‐PbMYB11 module as a key energy‐metabolism regulatory axis in pear fruit, elucidating the molecular basis of glucose‐dependent stone cell development and providing valuable genetic targets for fruit quality molecular breeding.

**FIGURE 7:**
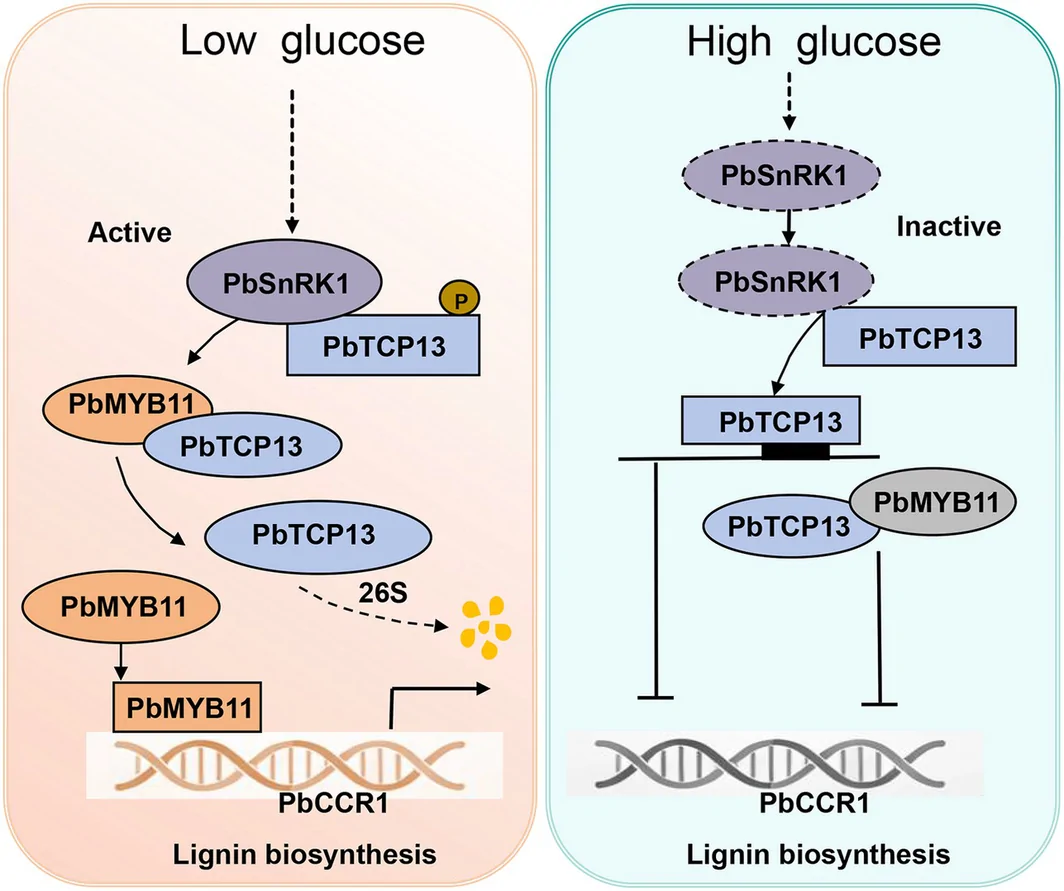
Working models for the glucose‐modulated PbSnRK1‐PbTCP13‐PbMYB11 molecular switch in pear. Under low‐glucose conditions, activated PbSnRK1 exhibits high kinase activity, which phosphorylates PbTCP13 at Ser265 and targets it for 26S proteasome‐mediated degradation. This degradation alleviates the transcriptional repression of PbMYB11, thereby activating the expression of the key lignin biosynthetic gene *PbCCR1* and promoting lignin biosynthesis. Under high‐glucose conditions, PbSnRK1 is inactivated and fails to phosphorylate PbTCP13, resulting in the accumulation of stable PbTCP13 protein. Accumulated PbTCP13 suppresses lignin biosynthesis through both direct transcriptional repression of *PbCCR1* and physical interaction with PbMYB11 to inhibit its activity.

## 4 Experimental Procedures

### 4.1 Plant Materials and Treatment

Seven high‐stone cell cultivars (Suli, Anli, Jianba, Yaguang, Huiyangsuan, Ruaner, Huangguan) and seven low‐stone cell cultivars (Cuiguan, Fengshui, Qiuyue, Zaosu, Aiganshui, Xingshui, Yuluxiang) were used in this study. All fruit samples were collected from the Pear Germplasm Repository of the National Pear Improvement Center (Jiangsu, China), Nanjing Agricultural University. Fruits were sampled at 15, 35, 55, 80 and 100 days after full bloom (DAFB), then immediately frozen in liquid nitrogen, and stored at −80°C until use. The ‘Suli’ (*P. bretschneideri*) pear fruits at 35 DAFB were used for transient transfection and allowed to recover for 24 h post‐infiltration. The fruits were then randomly divided into three groups and incubated at 25°C. Two groups were sprayed with approximately 1.5 mL per fruit of 1% or 4% (w/v) glucose solution containing 0.1% (v/v) Tween‐20 once every 24 h for 7 days, while the control group was sprayed with an equal volume of water containing 0.1% (v/v) Tween‐20. Following treatment, fruit tissues were harvested, immediately frozen in liquid nitrogen, and stored at −80°C until use.

Pear calli were induced from flesh tissue of European pear ( *P. communis* ) fruitlets and maintained on solid Murashige and Skoog (MS) medium in the dark under sterile conditions, with subculturing performed every 15 days. For glucose treatment, newly generated transgenic calli and wild‐type (WT) calli were cultured in the dark on sucrose‐free MS medium supplemented with 1% and 4% (w/v) glucose (Glc), respectively. Phenotypic observations were performed after 14 days of treatment, after which calli were collected after treatment and stored at −80°C until used.

*Arabidopsis thaliana* and *Nicotiana benthamiana* plants were grown in a greenhouse at 22°C under a 16 h light/8 h dark photoperiod with a light intensity of approximately 10 000 lx. For transient expression assays, the 4th to 6th leaves were selected from 4‐week‐old *N. benthamiana* plants.

For glucose treatment of Arabidopsis, surface‐sterilized seeds were germinated and grown on sucrose‐free MS medium supplemented with 0%, 1%, or 4% (w/v) glucose. Seedling phenotypes were recorded at 8 days after germination, and the seedlings were then transferred to soil. The plants were irrigated with water containing 1% or 4% (w/v) glucose once a week for four consecutive weeks; control plants were irrigated with an equal volume of water. At 8 weeks after germination, plant height was recorded, and the basal inflorescence stems were sampled for histological analysis.

### 4.2 Measurement of the Lignin and Stone Cell Content

Lignin content was determined following the acetyl bromide method as previously described (Tao et al. 2009). Briefly, dried samples of 0.01 g were homogenized in 95% ethanol until completely homogeneous, then washed three times with 95% ethanol and an ethanol‐hexane mixture (1:2, v/v), and subsequently dried. The dried precipitate was incubated in 2 mL of 25% (v/v) acetyl bromide solution at 70°C for 30 min. The reaction was terminated by the addition of 0.9 mL of 2 M NaOH and 0.1 mL of 7.5 M hydroxylamine hydrochloride. The resulting solution was diluted to a final volume of 10 mL with acetic acid, and absorbance was measured spectrophotometrically at 280 nm. Lignin content was calculated based on a calibration curve constructed using a lignin standard (Sigma‐Aldrich, USA).

Stone cell content was determined using the freezing‐HCl treatment method as previously described (Tao et al. 2009), and results were expressed as the dry weight (g) per pear fruit.

### 4.3 Determination of Soluble Sugar

Soluble sugar extraction from pear fruit tissues was performed according to a previously reported method (Wu et al. 2022). The contents of four major soluble sugar components, including fructose, sorbitol, glucose, and sucrose, were determined with an ACQUITY H‐Class UPLC system (Waters, USA).

### 4.4 RNA Isolation and Reverse‐Transcription Quantitative PCR

Total RNA was isolated using the RNA Isolator Total RNA Extraction Reagent (Vazyme, Nanjing, China). The isolated RNA (1 μg) was reverse‐transcribed into cDNA using HiScript II qRT SuperMix for qPCR (Vazyme) according to the manufacturer's instructions. Real‐time quantitative PCR analysis was performed using LightCycler 480 SYBR Green Master (Roche, USA). The cDNA was amplified with the primers listed in Table S2. *AtActin* and *PbUBQ* were used as internal reference genes for *Arabidopsis thaliana* and pear samples, respectively. The relative gene expression levels were calculated using the 2^−ΔΔCT^ method.

### 4.5 Site‐Directed Mutagenesis

Mutant variants were obtained using the Mut Express II Fast Mutagenesis Kit V2 (Vazyme). Site‐directed mutagenesis was performed according to the manufacturer's instructions, using the original plasmids as the template. The mutagenic primers are listed in Table S2. The resulting mutated plasmids were verified by sequencing.

### 4.6 Subcellular Localization Analysis

The full‐length coding sequences (CDS) without termination codons of *PbSnRK1*, *PbTCP13*, *PbTCP13* ^*S265A*^ and *PbMYB11* were inserted into the *Xba* I/*BamH* I sites of the pCAMBIA1300 vector to generate *Pro35S: PbSnRK1‐GFP*, *Pro35S: PbTCP13*‐*GFP*, *Pro35S: PbTCP13* ^*S265A*^ *‐GFP* and *Pro35S: PbMYB11‐GFP* constructs, respectively. *Agrobacterium tumefaciens* strain GV3101 containing the recombinant constructs or the 35S: GFP empty vector was infiltrated into the leaves of 5‐week‐old *N. benthamiana* plants. The infiltrated leaves were cultured in the dark for 48 h. Green fluorescent signals were observed using a confocal laser scanning microscope (LSM 780, Zeiss, Oberkochen, Germany). The primers used for vector construction are listed in Table S2.

### 4.7 Transient Transformation of Pear Fruits

For transient overexpression, the coding sequences (CDS) of *PbSnRK1*, *PbTCP13* and *PbMYB11* were inserted into the *pCAMBIA1300‐35S‐GFP* vector to generate *Pro35S: PbSnRK1‐GFP, Pro35S: PbTCP13‐GFP* and *Pro35S: PbMYB11‐GFP* constructs, respectively. For the virus‐induced gene silencing (VIGS) assay, gene fragments (200–300 bp) of *PbSnRK1*, *PbTCP13* and *PbMYB11* were inserted into the pTRV2 vector and transformed into *A. tumefaciens* strain GV3101. The nucleotide sequences and positions of all target fragments are listed in Table S5. The recombinant pTRV2 constructs or empty pTRV2 (negative control) were mixed with pTRV1 at a 1:1 ratio and co‐infiltrated into ‘Suli’ pear fruits at 35 DAFB using a needle syringe. After infiltration, the fruits were incubated in the dark for 24 h, followed by cultivation at 22°C under a 16 h light/8 h dark photoperiod for 7 days. The fruit tissues surrounding the injection sites were collected and stored at −80°C until use.

### 4.8 Stable Transformation of Arabidopsis

To generate Arabidopsis transgenic lines overexpressing *PbSnRK1*, *PbTCP13* and *PbMYB11*, *Agrobacterium* cells harbouring the *Pro35S: PbSnRK1‐GFP*, *Pro35S: PbTCP13‐GFP* and *Pro35S: PbMYB11‐GFP* constructs were resuspended in an infiltration solution (10% sucrose, 200 μM acetosyringone, 0.005% Silwet L‐77, OD₆₀₀ = 0.8, pH 5.7). Stable transformation of Arabidopsis ecotype Col‐0 was performed according to the previously described floral dip method (Clough and Bent 1998). Homozygous lines were selected over three successive generations on medium supplemented with 20 mg/L hygromycin. The primers used for the vector construction are listed in Table S2.

### 4.9 Pear Calli Transformation

Transgenic pear calli were generated as previously described (Bai et al. 2019). Briefly, pear calli were infected with *A. tumefaciens* strain GV3101 carrying *PbSnRK1*‐pCAMBIA1300, *PbTCP13*‐pCAMBIA1300, *PbMYB11*‐pCAMBIA1300, or the empty pCAMBIA1300 vector (control), and then co‐cultured on MS solid medium for 2 days in darkness. The positive pear calli were screened on solid MS medium supplemented with hygromycin (20 mg/L) and timentin (200 mg/L) under continuous darkness at 24°C for 1–3 months and subcultured every 3 weeks.

To generate the co‐expression calli lines (*PbTCP13*‐OE/*PbSnRK1*‐OE and *PbTCP13*‐OE/*PbMYB11*‐OE), the confirmed *PbSnRK1*‐OE or *PbMYB11*‐OE calli (generated with the pCAMBIA1300 vector, hygromycin resistance) were re‐transformed with *A. tumefaciens* GV3101 carrying *PbTCP13‐*pRI101 (kanamycin resistance). Double transformants were selected on MS medium supplemented with hygromycin (20 mg/L) and kanamycin (60 mg/L) and were further confirmed by qRT‐PCR analysis of both transgenes. The primers used for the vector construction are listed in Table S2.

### 4.10 Histological Analysis

For light microscopy analysis, the basal part of the primary inflorescence stems of 8‐week‐old Arabidopsis WT and *PbSnRK1*, *PbTCP13* and *PbMYB11* transgenic Arabidopsis were fixed in formalin‐acetic acid‐alcohol (FAA) fixative [1.9% formaldehyde solution (v/v), 5% glacial acetic acid (v/v), 63% ethyl alcohol (v/v), 30.1% water (v/v)] at 4°C for 1 week. Samples were dehydrated through a graded ethanol series and embedded in paraffin. Transverse sections of 5 μm thickness were cut using a Leica RM 2015 microtome (Leica Mikrosysteme, Germany). The sections were placed onto glass slides and stained with 1% (w/v) Toluidine Blue O [with 1% (w/v) sodium borate] for 5 min. After staining, the sections were observed using a Leica TCS SP2 spectral confocal microscope (Leica Microsystems, Germany).

### 4.11 Yeast Two‐Hybrid Assay (Y2H)

The full‐length CDSs of *PbSnRK1* and *PbMYB11* were cloned into the *EcoR* I/*BamH* I site of the pGBKT7 vector, while the CDS of *PbTCP13* was cloned into the *EcoR* I/*BamH* I site of the pGADT7 vector. The constructs were co‐transformed into the yeast strain AH109. Transformants were selected on SD/−Trp‐Leu medium and further screened on SD/−Trp‐Leu‐His‐Ade medium supplemented with 40 μg/mL X‐α‐gal. The plates were incubated at 30°C for 2–3 days to observe yeast colony growth. The primers used for vector construction are listed in Table S2.

### 4.12 GST Pull‐Down Assays

The full‐length CDSs of *PbSnRK1* and *PbMYB11* were cloned into the *BamH* I/*Hind* III sites of the pET‐32a vector and expressed in *E. coli* as Trx‐His fusions; the resulting full‐length His‐PbSnRK1 preparation did not reach the homogeneity required for in vitro kinase assays but was sufficient for the interaction analyses. The full‐length CDS of *PbTCP13* was cloned into the *BamH* I/*EcoR* I sites of the pGEX‐4 T‐1 vector. All primer sequences used for vector construction are listed in Table S2. For GST pull‐down assays, GST alone or GST‐PbTCP13 was immobilized on GST‐Trap agarose beads (Yeasen, Shanghai, China) and subsequently incubated with His‐PbSnRK1 at 4°C for 2 h with gentle agitation. Similarly, His‐PbMYB11 was incubated with GST alone or GST‐PbTCP13‐bound beads at 4°C for 2 h with gentle agitation. After washing to remove unbound protein, bound proteins were then eluted with glutathione, and the eluates were subjected to immunoblotting analysis using anti‐GST and anti‐His antibodies (Cowin Biotech, China, catalogue #CW0286M, 1:5000).

### 4.13 Luciferase Complementation Imaging Assays

The full‐length CDSs of *PbSnRK1* and *PbMYB11* were cloned into the *BamH* I/*Sal* I site of the pCAMBIA1300‐nLUC vector, while the CDS of *PbTCP13* and *PbTCP13* ^*S265A*^ were inserted into the *Kpn* I/*BamH* I sites of the pCAMBIA1300‐cLUC vector. These recombinant constructs were separately introduced into *A. tumefaciens* strain GV3101. The *Agrobacterium* cultures carrying the indicated constructs were co‐infiltrated into *N. benthamiana* leaves to generate interaction and negative control groups. After 48 h of incubation, the leaves were treated with 1 mmol/L of d‐luciferin substrate and incubated in the dark for 7 min. The luminescence signals were captured using a plant in vivo imaging system (CCD imaging system). The primer sequences used for vector construction are listed in Table S2.

### 4.14 Bimolecular Fluorescence Complementation Assay

The full‐length CDSs of *PbSnRK1* and *PbMYB11* were cloned into the *BamH* I/*Xho* I site of the YCE vector, while *PbTCP13* and *PbTCP13* ^*S265A*^ were inserted into the *BamH* I/*Xho* I sites of the YNE vector. *A. tumefaciens* strain GV3101 harbouring the gene‐YNE and gene‐YCE constructs was mixed in equal proportions and co‐infiltrated into *N. benthamiana* leaves. Leaves co‐infiltrated with empty YNE and YCE vectors were used as the negative control. After 48–72 h, suitable leaf samples were collected and mounted for observation. The fluorescence signals in the leaf were detected using a laser confocal scanning microscope (LSM510 Meta; Zeiss, Germany). The primers used for the vector construction are listed in Table S2.

### 4.15 Dual‐Luciferase Assay

The full‐length coding sequences of *PbSnRK1*, *PbTCP13*, *PbTCP13* ^*S265A*^ and *PbMYB11* were cloned into the *BamH* I/*EcoR* I site of the pGreenII 0029 62‐SK vector (gene‐SK), and a 2000‐bp fragment of the *PbCCR1* promoter was ligated into the *Hind* III/*BamH* I sites of the pGreenII 0800‐LUC vector (promoter‐LUC). All vectors were transformed into the *A. tumefaciens* strain GV3101 harbouring the pSoup vector. *A. tumefaciens* strain GV3101 containing the gene‐SK and promoter‐LUC was infiltrated into *N. benthamiana* leaves at a ratio of 1:9. At 24 h after infiltration, the infiltrated leaves were uniformly sprayed with 1% or 4% (w/v) glucose solutions, and the plants were further incubated for 48 h before the infiltrated leaf tissues were collected; leaves sprayed with water served as controls. LUC and REN activity were detected using a dual‐luciferase assay kit (Promega Corporation, Madison, WI, USA) and a luminometer (Molecular Devices, Sunnyvale, CA, USA). The transactivation activity of candidate genes was evaluated by the LUC/REN ratio. The primer sequences used for vector construction are listed in Table S2.

### 4.16 In Vitro Kinase Assay

The truncated PbSnRK1 (containing the S_TKc and UBA domains) was expressed as an MBP fusion and purified to homogeneity, following the strategy described for MdKIN10 (Zhi et al. 2026). In vitro phosphorylation assays were performed as previously described with minor modifications (Xu et al. 2024). Briefly, approximately 2 μg of His‐PbTCP13 and 1.5 μg of MBP‐PbSnRK1 were incubated in kinase reaction buffer (20 mM Tris–HCl [pH 7.5], 10 mM MgCl_2_, 0.1 mM CaCl_2_, 1 mM DTT and 10 mM ATP) with a total volume of 20 μL at 30°C for 2 h. The reaction was terminated by adding 5× SDS loading buffer, and the mixtures were boiled at 100°C for 5 min. Subsequently, the proteins were separated by 10% (w/v) SDS‐PAGE, and the levels of PbTCP13 and PbSnRK1 were detected by immunoblotting with anti‐His and anti‐MBP antibodies, respectively.

For phosphorylation detection, the reaction products were separated on an 8% SDS‐PAGE gel supplemented with 50 μM Phos‐tag and 100 μM manganese chloride, following the manufacturer's instructions (Cat. No. 304‐93521; Wako). After electrophoresis, the gel was washed three times with transfer buffer (50 mM Tris and 40 mM glycine) for 10 min each, with 10 mM EDTA supplemented into the buffer during the first two washes. The gel was then transferred to a PVDF membrane. An anti‐His antibody was used to detect the phosphorylation level of PbTCP13.

### 4.17 LC–MS/MS Analysis

Phosphorylated proteins were separated on a 10% SDS‐PAGE gel. After Coomassie Blue staining, the target bands were excised and destained. The dried protein pellets were incubated in 50 mM NH_4_HCO_3_ solution containing 25 mM CAA at room temperature for 30 min in the dark. After removing the above alkylation reagent, 1 μg trypsin was added for overnight enzymatic hydrolysis. The peptides were desalted using a C18 column and analysed by mass spectrometry. Phosphopeptides were detected using a TIMS‐TOF SCP high‐resolution mass spectrometer (Bruker). Data were analysed using PEAKS Studio version 13.0.

### 4.18 Cell‐Free Protein Degradation Assay

A cell‐free protein degradation assay was performed to examine the effect of PbSnRK1 on the stability of the PbTCP13 protein. Total protein extracts were obtained from pear calli overexpressing *PbSnRK1* using a degradation buffer (25 mM Tris–HCl pH 7.5, 10 mM NaCl, 10 mM MgCl_2_, 4 mM PMSF, 5 mM DTT and 10 mM ATP). Protein concentrations were determined using the Bradford assay with bovine serum albumin (BSA) as the standard. Protein extracts from calli transformed with the empty vector (GFP) were used as controls. Recombinant His‐PbTCP13 or His‐PbTCP13^S265A^ protein (10 μg) was mixed with total protein extracts (500 μg) in the presence of 50 μM MG132 or dimethyl sulfoxide (DMSO) as a solvent control, and incubated at 25°C for 0, 6, 12 and 24 h. The remaining levels of His‐PbTCP13 or His‐PbTCP13^S265A^ were analysed by SDS‐PAGE, and detected by immunoblotting using an anti‐His antibody.

### 4.19 Y1H Assays

The *PbCCR1* promoter fragment was cloned into the *Kpn*I/*Xho*I site of the pAbAi vector, while the CDSs of the transcription factor genes were cloned into the *EcoR* I/*BamH* I site of the pGADT7 vector (gene‐AD). The gene‐AD vectors were transformed into Y1HGold cells harbouring the pAbAi‐bait and screened on SD/−Leu/AbA medium. The primers used for the vector construction are listed in Table S2.

### 4.20 Electrophoretic Mobility Shift Assay

The EMSA was performed with His‐PbTCP13 and His‐PbMYB11 recombinant proteins. Sangon Biotech (Nanjing, China) synthesized two DNA probes from the *PbCCR1* promoter, containing the TGGGCT motif (for PbTCP13 EMSA) and the AC element (for PbMYB11 EMSA), respectively, and then labelled them with biotin. The competition probes were unlabeled DNA fragments, while the mutation probes were mutated at the key binding sites and were not biotinylated. The EMSA was performed as previously described (Gong et al. 2024). The EMSA probe sequences are listed in Table S2.

### 4.21 Sequence and Phylogenetic Analyses

Homologous sequences of *PbTCP13* and *PbMYB11* were identified from the National Center for Biotechnology Information (https://blast.ncbi.nlm.nih.gov/Blast.cgi) by BLASTP searches using the respective protein sequences as queries. Multiple sequence alignments were conducted with Jalview software (http://www.jalview.org). Phylogenetic trees were constructed using the neighbour‐joining method in MEGA 5.0 with 1000 bootstrap replicates.

### 4.22 RNA‐Seq Data Analysis

Library construction and sequencing were performed by Ling'en Biotechnology (Shanghai, China) on an Illumina NovaSeq platform. Clean data were obtained from the raw data by eliminating low‐quality reads and reads containing adapter sequences or polyadenylated tails. The filtered reads were subsequently mapped to the *P. communis* reference genome. The expression levels of genes were quantified using the Fragments Per Kilobase of transcript per Million mapped reads (FPKM) metric. Differentially expressed genes (DEGs) were identified by a differential gene expression analysis tool, TBtools‐II. The obtained *p*‐values were adjusted based on the Benjamini‐Hochberg method, and genes with adjusted *p*‐values < 0.05 and |log_2_ fold change| ≥ 1 were identified as differentially expressed genes.

### 4.23 Statistical Analysis

All data in this study were analysed using a two‐tailed Student's *t*‐test or one‐way analysis of variance (ANOVA) to determine statistical significance with GraphPad Prism v.9.0.

## Author Contributions

S.T. was responsible for designing the research protocol. S.T., J.H. and X.W. jointly conceived and designed the experimental protocol. J.H. performed most of the experimental procedures. X.W. and W.L. assisted with part of the experimental work. W.Z., J.C., M.N., X.Y. and Y.Y. participated in the data analysis. J.H. and S.T. were responsible for drafting the manuscript. S.Z., K.Q. and Z.X. prepared the plant materials and provided technical assistance. A.B., K.S. (Shiratake) and K.S. (Khanizadeh) assisted with revising the manuscript. All authors reviewed and approved the final manuscript.

## Funding

This work was supported by the Jiangsu provincial Key Research and Development program (BE2023365), the Postgraduate Research & Practice Innovation Program of Jiangsu Province (KYCX24‐0969), the Earmarked Fund for China Agriculture Research System (CARS‐28), and the National Natural Science Foundation of China (32573001).

## Conflicts of Interest

The authors declare no conflicts of interest.

## Supporting information


**Figure S1:** Glucose regulates lignin biosynthesis in pear fruit calli (Supports Figure 1). (a) Phenotype of pear fruit calli under different glucose concentrations (scale bar, 2 cm) and (b) lignin content. (c‐j) Expression levels of PbC3H1, PbC4H1, PbCCoAOMT, PbCCR1, PbCCR2, PbCSE1, PbHCT17 and PbPOD1 in pear fruit calli under different glucose treatments. (k) Phenotype of pear fruit calli treated with 0% Glc, Man and 4% Glc; (l) lignin content; (m‐t) expression levels of lignin biosynthetic genes. Data are presented as means ± SE (*n* = 3). Significant differences were determined using two‐tailed Student's *t*‐test (*p < 0.05; **p < 0.01). Different lowercase letters indicate significant differences determined by one‐way ANOVA (*p* < 0.05).
**Figure S2:** PbSnRK1 mediates glucose‐induced lignin biosynthesis (Supports Figure 1). (a) Expression levels of PbSnRK1 in pear fruit calli under different glucose treatments. (b) Phenotype of pear fruit calli treated with 0% Glc, 4% Glc and 4% Glc + T6P; (c) lignin content; (d‐k) expression levels of lignin biosynthetic genes. Data are presented as means ± SE (*n* = 3). Statistical significance was determined by two‐tailed Student's *t*‐test relative to the 0% glucose treatment (*p < 0.05; **p < 0.01).
**Figure S3:** Glucose is tightly coupled with stone cell lignification (Supports Figure 1). (a‐c) Heatmaps showing (a) lignin content, (b) stone cell content and (c) glucose content across 14 pear cultivars with divergent stone cell traits at five developmental stages (15 to 100 days after full bloom, DAFB). Cultivars were divided into high‐stone‐cell and low‐stone‐cell groups (7 cultivars per group). (d–f) Correlation analysis between glucose and lignin contents in the (d) full population, (e) high‐stone‐cell subgroup and (f) low‐stone‐cell subgroup. (g–i) Correlation analysis between lignin content and (g) fructose, (h) sucrose and (i) sorbitol contents in the full population. The correlations in (d–f) (glucose), (g) (fructose) and (i) (sorbitol) were statistically significant (*p* < 0.05), whereas that in (h) (sucrose) was not significant (*p* = 0.1340).
**Figure S4:** Lignin accumulation and expression of lignin biosynthetic genes in pear fruits under different treatments (Supports Figure 1). (a) Lignin content in ‘Suli’ pear fruits with transient overexpression (OE) or virus‐induced gene silencing (VIGS) of PbSnRK1 under normal growth conditions (no exogenous glucose). (b‐e) RT‐qPCR analysis of lignin biosynthetic genes in pear fruits with transient PbSnRK1 OE or VIGS under (b, c) 4% or (d, e) 1% glucose treatment. Data are presented as means ± SE (*n* = 3). Significant differences were determined using two‐tailed Student's *t*‐test (*p < 0.05; **p < 0.01).
**Figure S5:** Kinase activity of PbSnRK1 is required for glucose‐dependent bidirectional regulation of lignin biosynthesis (Supports Figure 1). (a) Sanger sequencing confirmed the successful point mutation of Thr^175^ to Ala in PbSnRK1, with the mutated nucleotides highlighted in red boxes. (b) Lignin content in empty vector overexpression (EV‐OE), PbSnRK1‐OE and PbSnRK1T175A‐OE pear calli under normal growth conditions (no exogenous glucose). (c, d) RT‐qPCR analysis of lignin biosynthetic genes in EV‐OE, PbSnRK1‐OE and PbSnRK1T175A‐OE pear calli under (c) 4% or (d) 1% glucose treatment. Data are presented as means ± SE (*n* = 3). Significant differences were determined using two‐tailed Student's *t*‐test (*p < 0.05; **p < 0.01).
**Figure S6:** Heterologous expression of PbSnRK1 regulates lignin biosynthesis and secondary cell wall thickness in Arabidopsis (Supports Figure 1). (a) Transcript levels of PbSnRK1 in wild‐type Arabidopsis Col‐0 (WT) and PbSnRK1‐OE transgenic lines. (b, e) Phenotype of 8‐day‐old PbSnRK1‐OE seedlings grown on MS medium with (b) 4% or (e) 1% (w/v) glucose (scale bar, 5 mm). (c, f) Plant height of 8‐week‐old WT and PbSnRK1‐OE plants under (c) 4% or (f) 1% glucose treatment (scale bar, 5 cm). (d, g) Expression levels of secondary cell wall (SCW) biosynthetic genes under (d) 4% or (g) 1% glucose treatment. (h) Lignin content in WT and PbSnRK1‐OE Arabidopsis under glucose‐free conditions. (i, j) SCW thickness of vessel cells under (i) 4% or (j) 1% glucose treatment. Data are presented as means ± SE (*n* = 3 for a, d, g, h; *n* = 13 for i, j). Significant differences were determined using two‐tailed Student's *t*‐test (n.s., no significance, *p* > 0.05; *p < 0.05; **p < 0.01).
**Figure S7:** Identification of PbTCP13 (Supports Figures 2 and 3). (a) Phylogenetic analysis of the TCP family from various plants. TCP family protein sequence derived from pear (Pb, Pyrus bretschneideri), Arabidopsis (At, *Arabidopsis thaliana* ) and apple (Md, Malus × domestica). Different subgroups are marked with different colours, respectively. (b) Phylogenetic analysis of PbTCP13. (c) Expression levels of PbTCP13 in pear fruit calli under 0%, 1%, 2%, 3%, 4% or 6% (w/v) glucose treatment. (d) Subcellular localization showed that PbTCP13 was localized in the nucleus of N. benthamiana leaf cells (scale bar, 20 μm). (e) Multiple sequence alignment of PbTCP13. The Ser265 is marked with red box. Data are presented as means ± SE (*n* = 3). Significant differences were determined using two‐tailed Student's *t*‐test (*p < 0.05; **p < 0.01).
**Figure S8:** Functional characterization of PbTCP13 in the regulation of lignin biosynthesis (Supports Figure 2). (a, b) Expression levels of lignin biosynthetic genes in pear fruits with transient (a) PbTCP13‐OE or (b) PbTCP13‐VIGS under 4% glucose treatment and (c) lignin content under normal growth conditions (without exogenous glucose). (d) Expression levels of lignin biosynthetic genes in EV‐OE and PbTCP13‐OE pear calli under 4% glucose treatment and (e) lignin content under normal growth conditions. (f) RT‐qPCR analysis of PbTCP13 expression levels in WT and two independent PbTCP13‐OE transgenic Arabidopsis lines. (g) Phenotype of 8‐day‐old PbTCP13‐OE seedlings grown on MS medium with 4% glucose (scale bar, 5 mm). (h) Plant height of 8‐week‐old WT and PbTCP13‐OE Arabidopsis plants under 4% glucose treatment (scale bar, 5 cm). (i) Expression levels of SCW biosynthetic genes under 4% glucose treatment. (j) Lignin content under glucose‐free conditions. (k) Secondary cell wall thickness of vessel cells under 4% glucose treatment. Data are presented as means ± SE (*n* = 3 for a‐f, i, j; *n* = 13 for k). Significant differences were determined using two‐tailed Student's *t*‐test (n.s., no significance, *p* > 0.05; *p < 0.05; **p < 0.01).
**Figure S9:** Identification of transcripts by weighted gene co‐expression network analysis (WGCNA) of PbSnRK1‐OE and EV‐OE pear calli under 1% or 4% glucose treatment (Supports Figure 4). Module‐trait correlations and the corresponding *p*‐values are in parentheses. The expression level of lignin‐related structural genes functions as trait data. The left panel presents the 10 modules. The colour scale on the right presents the module–trait correlations from‐1 (green) to 1 (red).
**Figure S10:** Characterization of PbMYB11 and its expression pattern (Supports Figure 4). (a) Expression levels of six MYB TFs in the ‘plum3’ module of PbSnRK1‐OE pear calli under 1% treatment. (b) Phylogenetic analysis of PbMYB11. (c) Amino acid sequence alignment of PbMYB11 with homologous R2R3‐MYB genes. The R2R3 DNA‐binding domain is highlighted in red boxes. (d) Expression levels of PbMYB11 in PbSnRK1‐OE and PbTCP13‐OE pear calli under 4% glucose treatment. Data are presented as means ± SE (*n* = 3). Significant differences were determined using two‐tailed Student's *t*‐test (n.s., no significance, *p* > 0.05; **p < 0.01).
**Figure S11:** Ser265 phosphorylation regulates PbTCP13‐PbMYB11 interaction and PbMYB11 activation of PbCCR1 (Supports Figure 4). (a) Subcellular localization showed that PbTCP13S265A was localized in the nucleus of N. benthamiana leaf cells (scale bar, 20 μm). (b) BiFC and (c) LCI assays revealed that the S265A mutation in PbTCP13 slightly attenuated the interaction intensity between PbTCP13 and PbMYB11 in N.benthamiana leaf cells (scale bar, 20 μm). (d) Dual‐luciferase reporter assays verified that PbMYB11 significantly activates the transcriptional activity of the PbCCR1 promoter in N. benthamiana leaf cells. Data are presented as means ± SE (*n* = 6). Significant differences were determined using two‐tailed Student's *t*‐test (**p < 0.01).
**Figure S12:** Lignin accumulation and lignin biosynthetic gene expression in PbMYB11‐OE pear fruits under different glucose treatments (Supports Figure 5). (a, b) Expression levels of lignin biosynthetic genes in pear fruits with transient (a) PbMYB11‐OE or (b) PbMYB11‐VIGS under 1% glucose treatment. (c) Phloroglucinol‐HCl staining of EV‐OE, PbMYB11‐OE, EV‐VIGS and PbMYB11‐VIGS transgenic pear fruits following 4% glucose treatment for 7 days (scale bar, 0.5 cm). (d) Lignin content and (e) stone cell content in the above transgenic pear fruits. (f) Lignin content in the above transgenic pear fruits under normal growth conditions (no exogenous glucose). (g, h) RT‐qPCR analysis of lignin biosynthetic genes in pear fruits with transient (g) PbMYB11‐OE or (h) PbMYB11‐VIGS under 4% glucose treatment. Data are presented as means ± SE (*n* = 3). Significant differences were determined using two‐tailed Student's *t*‐test (n.s., no significance, *p* > 0.05; *p < 0.05; **p < 0.01).
**Figure S13:** Lignin accumulation and lignin biosynthetic gene expression in PbMYB11‐OE pear calli under different conditions (Supports Figure 5). (a) Expression levels of lignin biosynthetic genes in EV‐OE and PbMYB11‐OE pear calli under 1% glucose treatment. (b) Phloroglucinol‐HCl staining of EV‐OE and PbMYB11‐OE transgenic pear calli following 4% glucose treatment (scale bar, 0.5 cm). (c) Lignin content in the above transgenic pear calli. (d) RT‐qPCR analysis of lignin biosynthetic genes in the above transgenic pear calli. (e) Lignin content in EV‐OE and PbMYB11‐OE transgenic pear calli under normal growth conditions (no exogenous glucose). Data are presented as means ± SE (*n* = 3). Significant differences were determined using two‐tailed Student's *t*‐test (n.s., no significance, *p* > 0.05; *p < 0.05; **p < 0.01).
**Figure S14:** Heterologous expression of PbMYB11 regulates lignin biosynthesis in Arabidopsis (Supports Figure 5). (a) Expression levels of PbMYB11 in WT and two independent PbMYB11‐OE transgenic Arabidopsis lines. (b) Phenotype of 8‐d‐old PbMYB11‐OE seedlings grown on MS medium with 1% glucose (scale bar, 5 mm). (c) Plant height of 8‐week‐old WT and PbMYB11‐OE Arabidopsis plants under 1% glucose treatment (scale bar, 5 cm). (d) Expression levels of lignin biosynthetic genes under 1% glucose treatment. (e) Lignin content under glucose‐free conditions. (f) SCW thickness of vessel cells under 1% glucose treatment. Data are presented as means ± SE (*n* = 3 for a, d, e; *n* = 13 for f). Significant differences were determined using two‐tailed Student's *t*‐test (n.s., no significance, *p* > 0.05; *p < 0.05; **p < 0.01).
**Figure S15:** Lignin accumulation and gene expression of the PbSnRK1‐PbTCP13‐PbMYB11 module under different glucose treatments (Supports Figure 6). (a) Lignin content in EV‐OE, PbSnRK1‐OE, PbTCP13‐OE, PbTCP13‐OE/PbSnRK1‐OE, PbMYB11‐OE and PbTCP13‐OE/PbMYB11‐OE transgenic pear calli under 4% glucose treatment. (b) RT‐qPCR analysis of lignin biosynthetic genes in EV‐OE, PbSnRK1‐OE, PbTCP13‐OE, PbTCP13‐OE/PbSnRK1‐OE, PbMYB11‐OE and PbTCP13‐OE/PbMYB11‐OE transgenic pear calli under 1% glucose treatment. (c) RT‐qPCR analysis of lignin biosynthetic genes in EV‐OE, PbSnRK1‐OE, PbTCP13‐OE and PbMYB11‐OE transgenic pear fruits under 1% glucose treatment. (d) RT‐qPCR analysis of lignin biosynthetic genes in EV‐VIGS, PbTCP13‐VIGS, PbSnRK1‐OE/PbTCP13‐VIGS, PbMYB11‐VIGS and PbMYB11‐VIGS/PbTCP13‐VIGS transgenic pear fruits under 1% glucose treatment. (e, f) Lignin content in the above transgenic pear fruit lines under 4% glucose treatment. Data are presented as means ± SE (*n* = 3). Significant differences were determined using two‐tailed Student's *t*‐test (n.s., no significance, *p* > 0.05; *p < 0.05; **p < 0.01).

**Table S1:** FPKM values and annotations of genes in the ‘plum3’ module.

**Table S2:** List of primers used in this study.

**Table S3:** Correlation analysis between transcript abundance of glucose sensor genes and lignin accumulation in glucose‐treated pear calli.

**Table S4:** Candidate interactors identified by Y2H screening using PbSnRK1 as bait.

**Table S5:** Nucleotide sequences of pTRV2 VIGS constructs.

## Data Availability

The data underlying this article are available in the NCBI Sequence Read Archive at http://www.ncbi.nlm.nih.gov/sra/, and can be accessed with the BioProject ID: PRJNA1456992. The previously published transcriptome datasets from 0% (control) and 4% glucose‐treated pear calli analysed in this study are available in the NCBI Sequence Read Archive under BioProject ID: PRJNA801923. The genes in this study have the following IDs: *PbSnRK1*, XM_009362316; *PbTCP13*, XM_048581676; *PbMYB11*, XM_009369374. All data are available in the main text or the Supporting Information (Figures S1–S15; Tables S1–, S5).
