# Supplementary material for: PbSnRK1‐Mediated Phosphorylation of PbTCP13 Regulates Glucose‐Responsive Lignin Biosynthesis via PbMYB11 During Pear Stone Cell Formation
Source: Plant Biotechnol J. 2026 Oct 15:10.1111/pbi.70770. Online ahead of print. doi: 10.1111/pbi.70770 (PMC13669904; doi:10.1111/pbi.70770)
Supplement: Supplementary file 1 [file PBI-9999-0-s006.pdf]

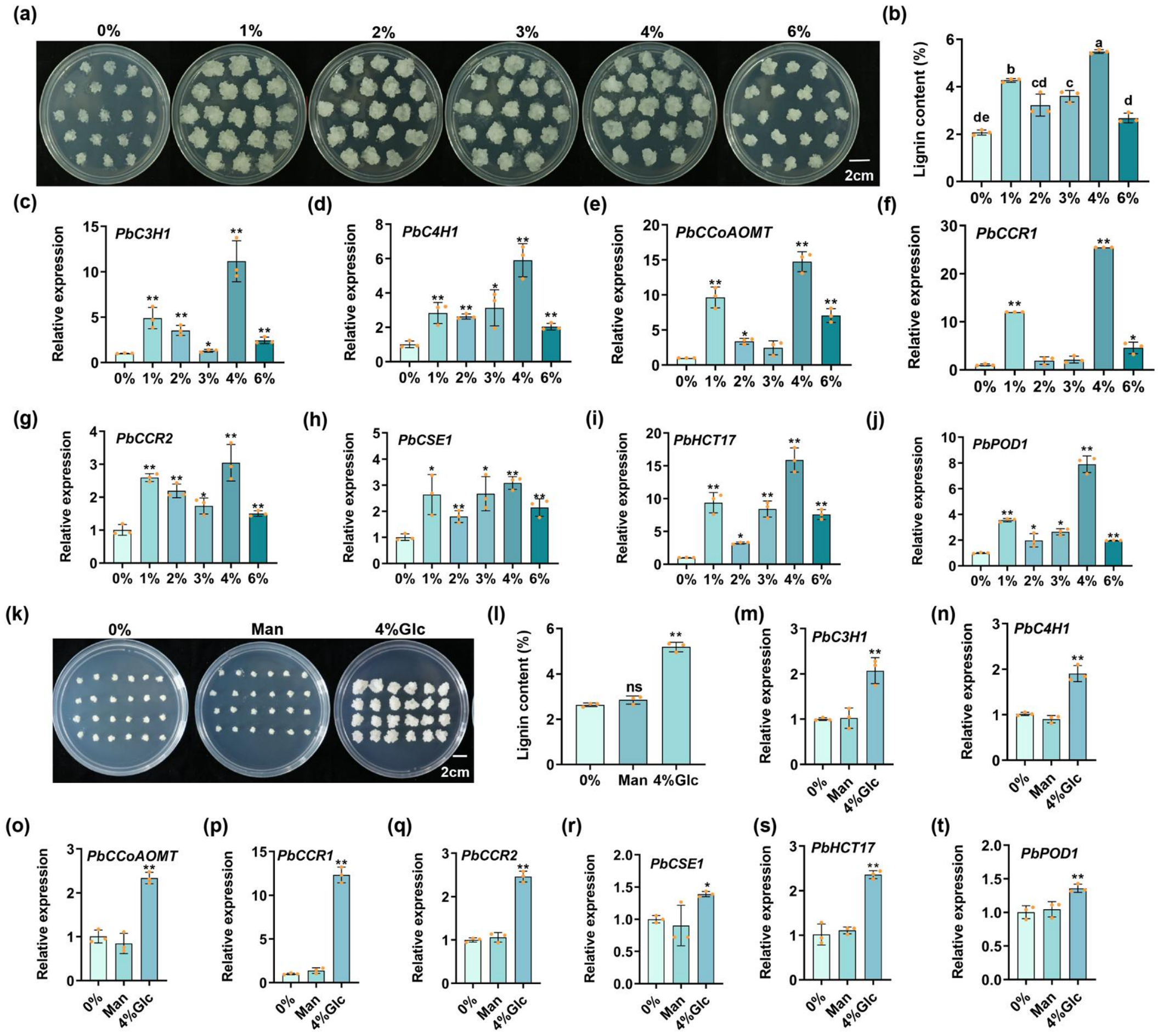

Figure S1 Glucose regulates lignin biosynthesis in pear fruit calli (Supports Figure 1). (a) Phenotype of pear fruit calli under different glucose concentrations (scale bar, 2 cm) and (b) lignin content. (c-j) Expression levels of *PbC3H1*, *PbC4H1*, *PbCCoAOMT*, *PbCCR1*, *PbCCR2*, *PbCSE1*, *PbHCT17*, and *PbPOD1* in pear fruit calli under different glucose treatments. (k) Phenotype of pear fruit calli treated with 0% Glc, Man, and 4% Glc; (l) lignin content; (m-t) expression levels of lignin biosynthetic genes. Data are presented as means ± SE (n = 3). Significant differences were determined using two-tailed Student's t-test (*P < 0.05; **P < 0.01). Different lowercase letters indicate significant differences determined by one-way ANOVA (P < 0.05).

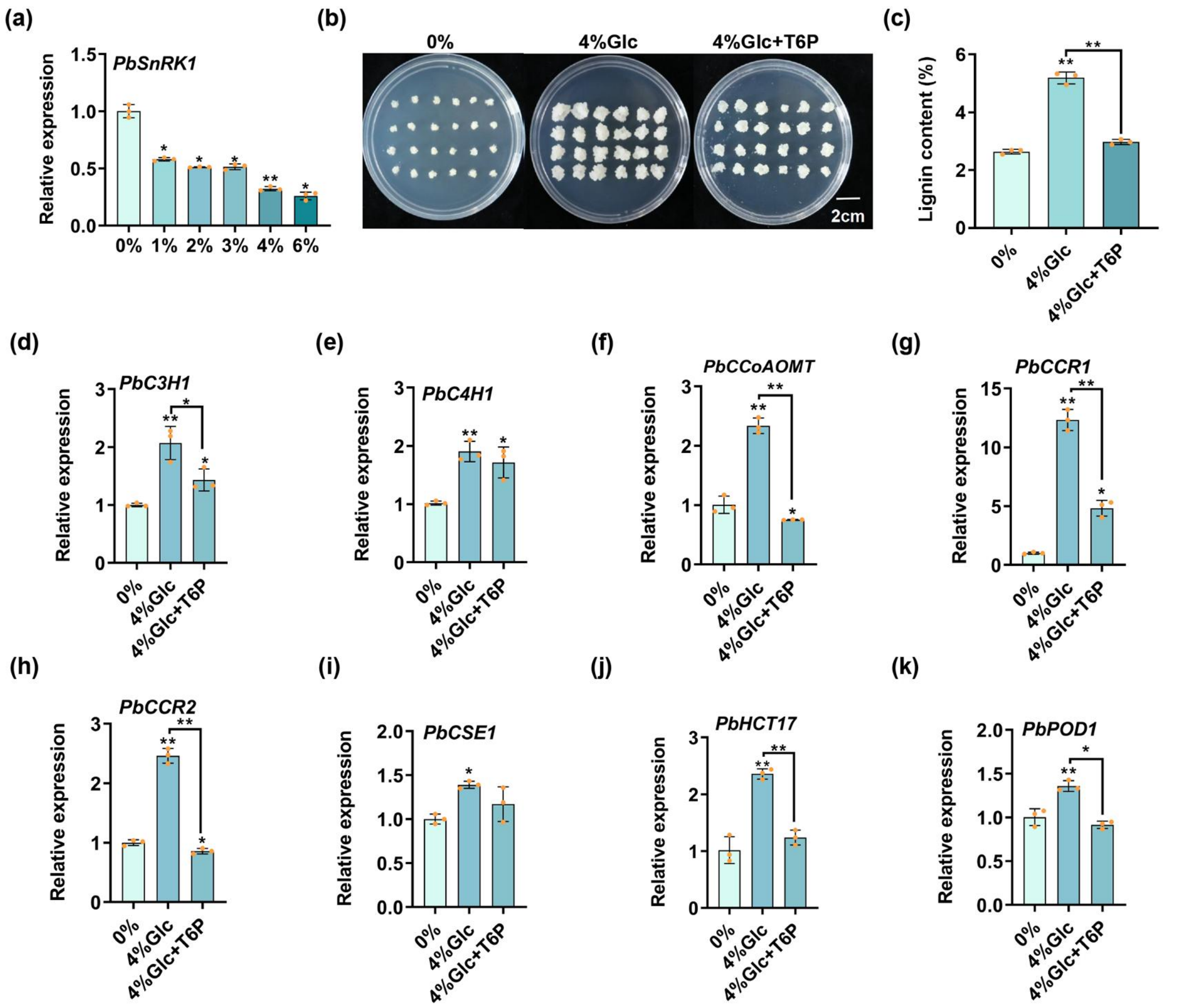

Figure S2 *PbSnRK1* mediates glucose-induced lignin biosynthesis (Supports Figure 1). (a) Expression levels of *PbSnRK1* in pear fruit calli under different glucose treatments. (b) Phenotype of pear fruit calli treated with 0% Glc, 4% Glc, and 4% Glc + T6P; (c) lignin content; (d-k) expression levels of lignin biosynthetic genes. Data are presented as means ± SE (n = 3). Statistical significance was determined by two-tailed Student's t-test relative to the 0% glucose treatment (*P < 0.05; **P < 0.01).

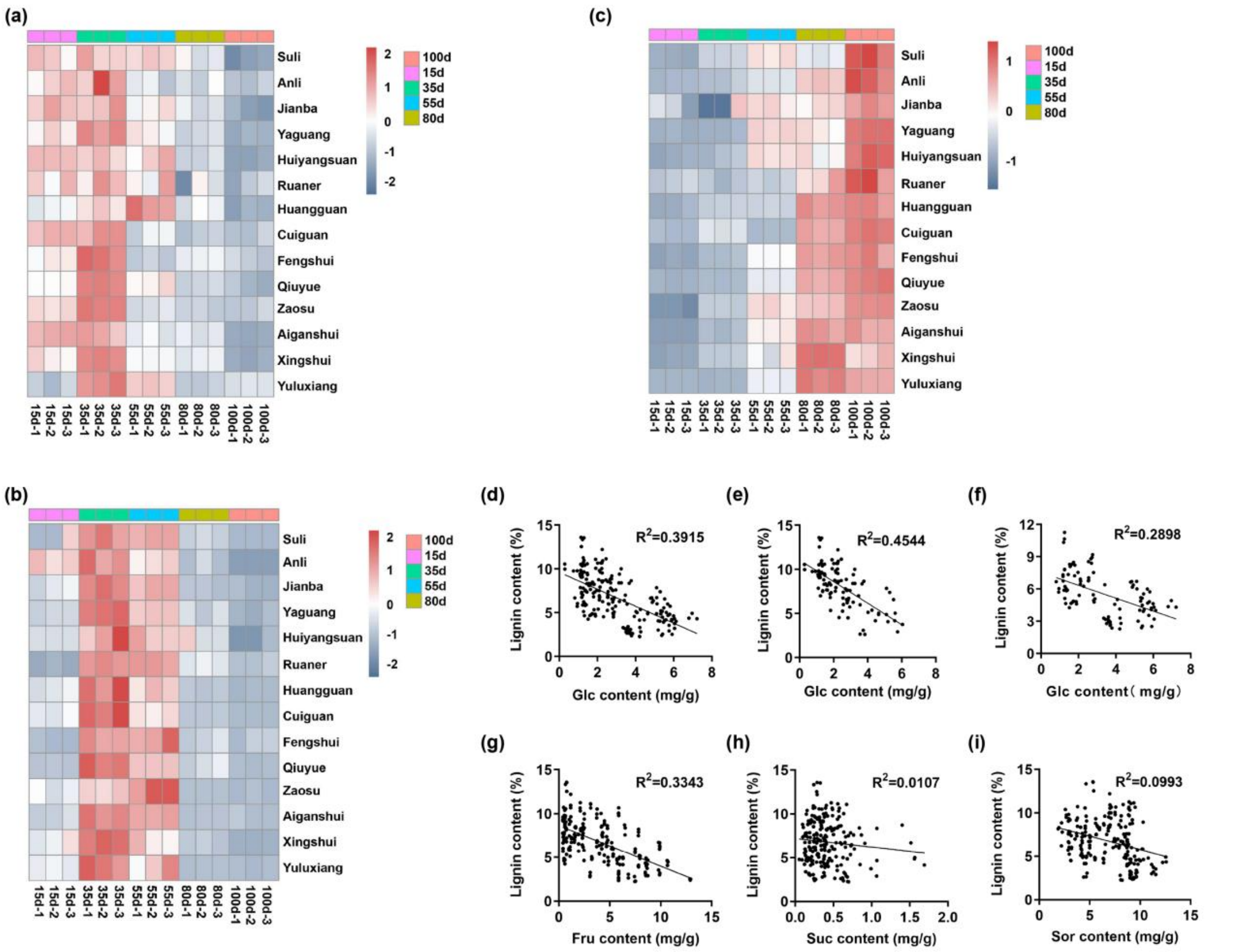

Figure S3 Glucose is tightly coupled with stone cell lignification (Supports Figure 1). (a-c) Heatmaps showing (a) lignin content, (b) stone cell content, and (c) glucose content across 14 pear cultivars with divergent stone cell traits at five developmental stages (15 to 100 days after full bloom, DAFB). Cultivars were divided into high-stone-cell and low-stone-cell groups (7 cultivars per group). (d-f) Correlation analysis between glucose and lignin contents in the (d) full population, (e) high-stone-cell subgroup, and (f) low-stone-cell subgroup. (g-i) Correlation analysis between lignin content and (g) fructose, (h) sucrose, and (i) sorbitol contents in the full population. The correlations in (d-f) (glucose), (g) (fructose) and (i) (sorbitol) were statistically significant ($P < 0.05$), whereas that in (h) (sucrose) was not significant ($P = 0.1340$).

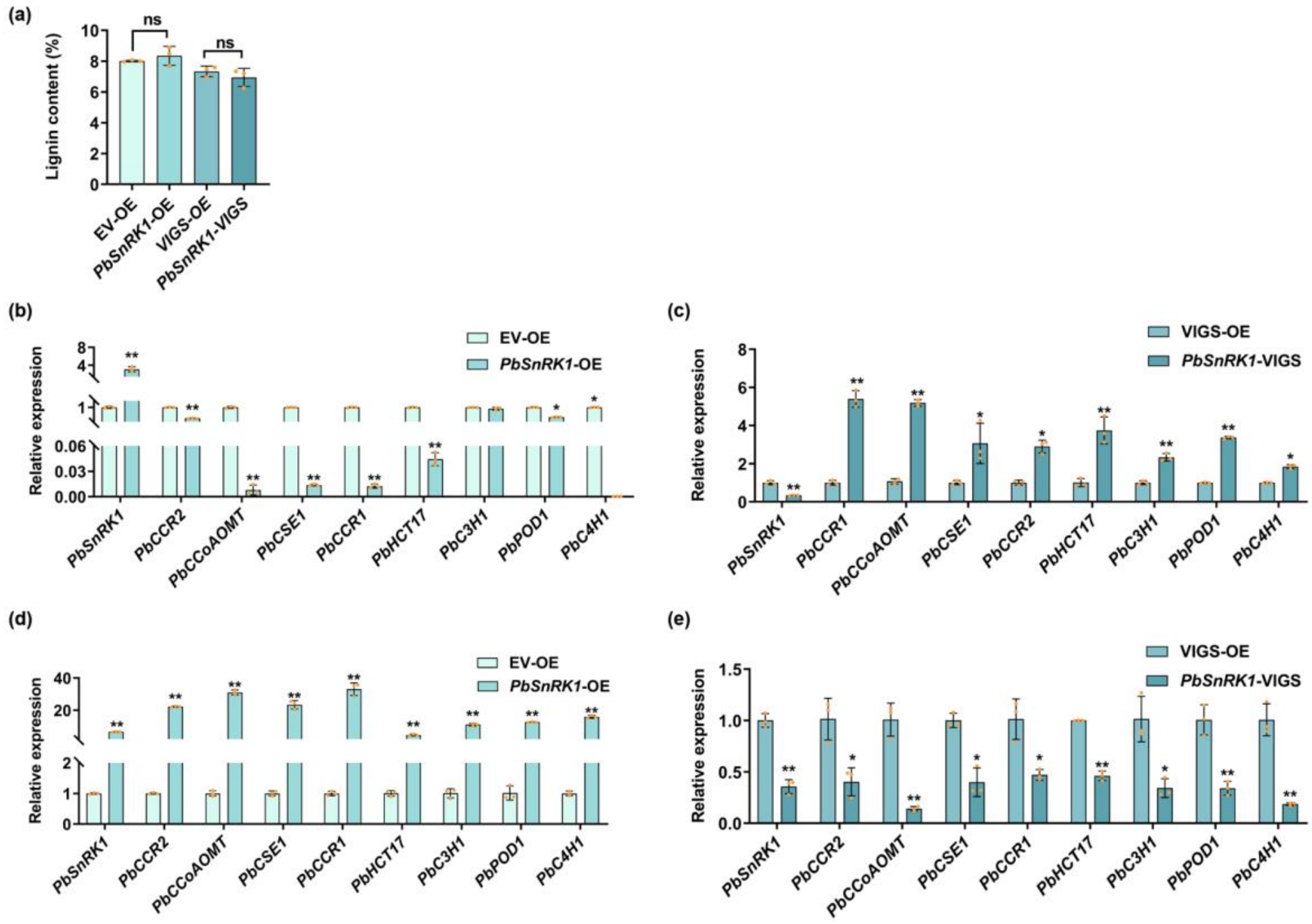

Figure S4 Lignin accumulation and expression of lignin biosynthetic genes in pear fruits under different treatments (Supports Figure 1). (a) Lignin content in 'Suli' pear fruits with transient overexpression (OE) or virus-induced gene silencing (VIGS) of *PbSnRK1* under normal growth conditions (no exogenous glucose). (b-e) RT-qPCR analysis of lignin biosynthetic genes in pear fruits with transient *PbSnRK1* OE or VIGS under (b,c) 4% or (d,e) 1% glucose treatment. Data are presented as means ± SE (n = 3). Significant differences were determined using two-tailed Student's t-test (**P* < 0.05; ***P* < 0.01).

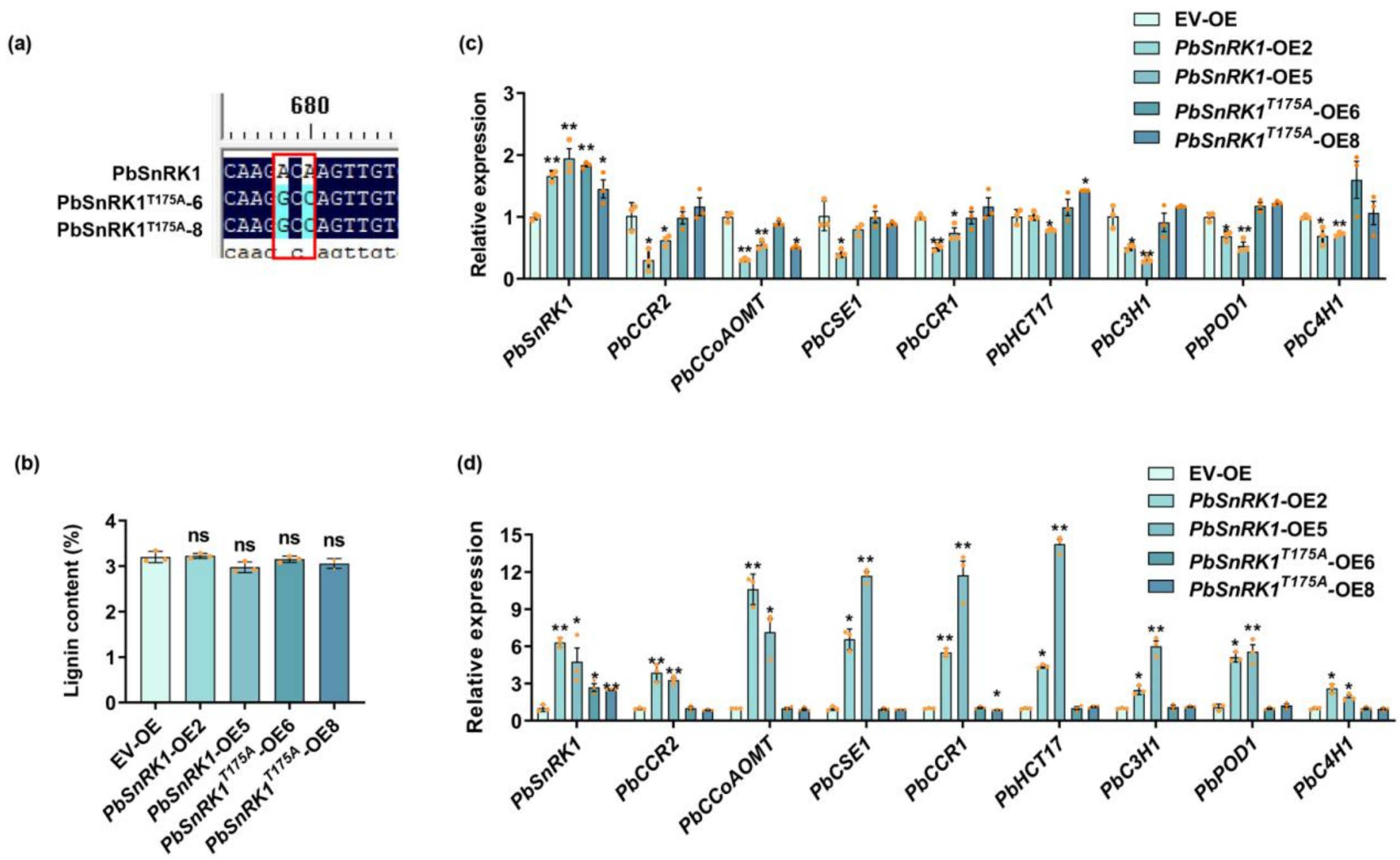

Figure S5 Kinase activity of *PbSnRK1* is required for glucose-dependent bidirectional regulation of lignin biosynthesis (Supports Figure 1). (a) Sanger sequencing confirmed the successful point mutation of Thr^{175} to Ala in PbSnRK1, with the mutated nucleotides highlighted in red boxes. (b) Lignin content in empty vector overexpression (EV-OE), *PbSnRK1*-OE, and *PbSnRK1*^{T175A}-OE pear calli under normal growth conditions (no exogenous glucose). (c, d) RT-qPCR analysis of lignin biosynthetic genes in EV-OE, *PbSnRK1*-OE, and *PbSnRK1*^{T175A}-OE pear calli under (c) 4% or (d) 1% glucose treatment. Data are presented as means ± SE (n = 3). Significant differences were determined using two-tailed Student's t-test (*$P < 0.05$; **$P < 0.01$).

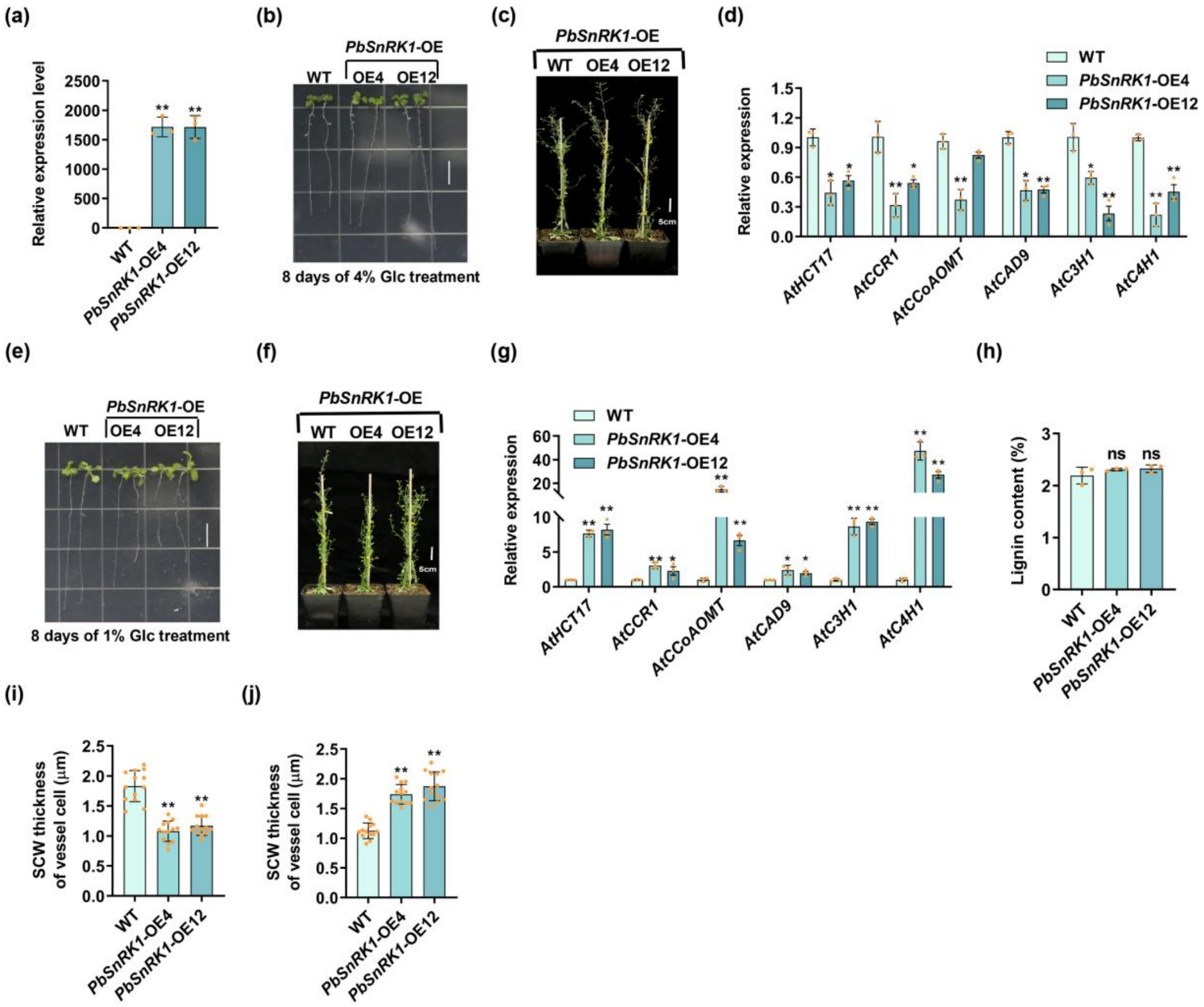

Figure S6 Heterologous expression of *PbSnRK1* regulates lignin biosynthesis and secondary cell wall thickness in Arabidopsis (Supports Figure 1). (a) Transcript levels of *PbSnRK1* in wild-type Arabidopsis Col-0 (WT) and *PbSnRK1*-OE transgenic lines. (b,e) Phenotype of 8-d-old *PbSnRK1*-OE seedlings grown on MS medium with (b) 4% or (e) 1% (w/v) glucose (scale bar, 5 mm). (c,f) Plant height of 8-week-old WT and *PbSnRK1*-OE plants under (c) 4% or (f) 1% glucose treatment (scale bar, 5 cm). (d,g) Expression levels of secondary cell wall (SCW) biosynthetic genes under (d) 4% or (g) 1% glucose treatment. (h) Lignin content in WT and *PbSnRK1*-OE Arabidopsis under glucose-free conditions. (i,j) SCW thickness of vessel cells under (i) 4% or (j) 1% glucose treatment. Data are presented as means ± SE (n = 3 for a, d, g, h; n = 13 for i, j). Significant differences were determined using two-tailed Student's t-test (n.s., no significance, $P > 0.05$; $^{*}P < 0.05$; $^{**}P < 0.01$).

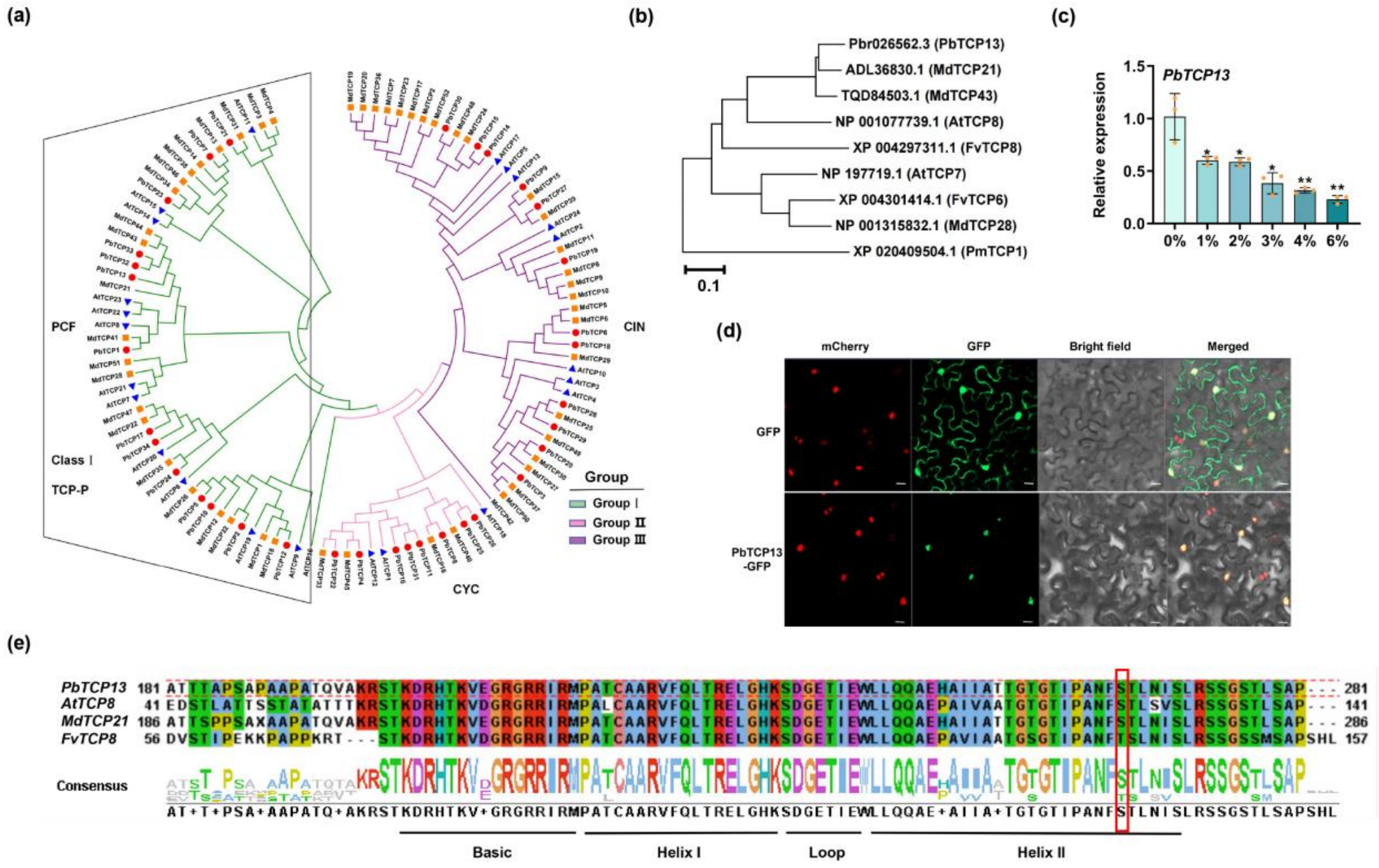

Figure S7 Identification of *PbTCP13* (Supports Figures 2 and 3). (a) Phylogenetic analysis of the TCP family from various plants. TCP family protein sequence derived from pear (Pb, *Pyrus bretschneideri*), Arabidopsis (At, *Arabidopsis thaliana*), and apple (Md, *Malus* × *domestica*). Different subgroups are marked with different colors, respectively. (b) Phylogenetic analysis of PbTCP13. (c) Expression levels of *PbTCP13* in pear fruit calli under 0%, 1%, 2%, 3%, 4%, or 6% (w/v) glucose treatment. (d) Subcellular localization showed that PbTCP13 was localized in the nucleus of *N. benthamiana* leaf cells (scale bar, 20 μm). (e) Multiple sequence alignment of *PbTCP13*. The Ser265 is marked with red box. Data are presented as means ± SE (n = 3). Significant differences were determined using two-tailed Student's t-test (**P* < 0.05; ***P* < 0.01).

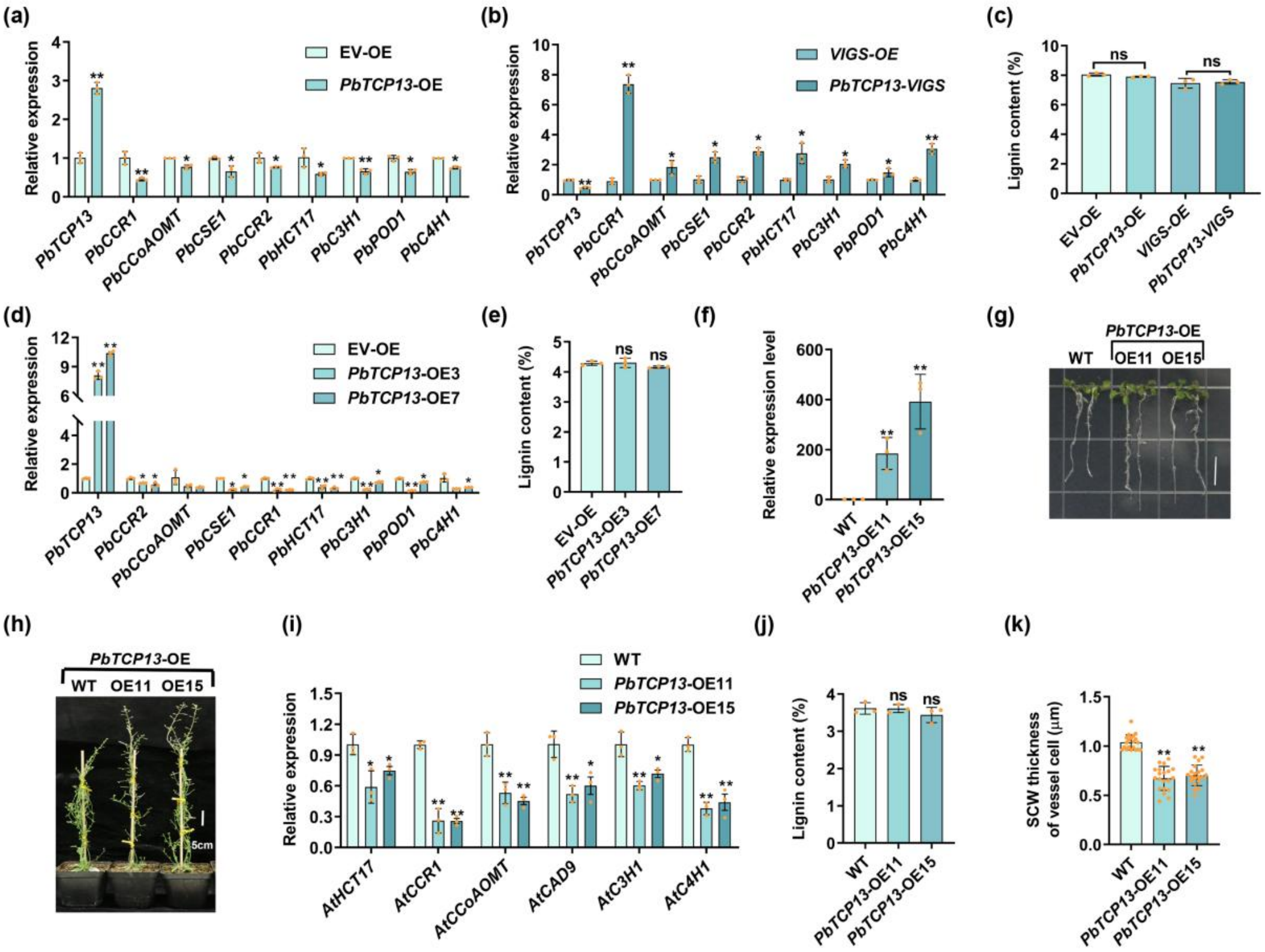

Figure S8 Functional characterization of *PbTCP13* in the regulation of lignin biosynthesis (Supports Figure 2). (a, b) Expression levels of lignin biosynthetic genes in pear fruits with transient (a) *PbTCP13*-OE or (b) *PbTCP13*-VIGS under 4% glucose treatment, and (c) lignin content under normal growth conditions (without exogenous glucose). (d) Expression levels of lignin biosynthetic genes in EV-OE and *PbTCP13*-OE pear calli under 4% glucose treatment, and (e) lignin content under normal growth conditions. (f) RT-qPCR analysis of *PbTCP13* expression levels in WT and two independent *PbTCP13*-OE transgenic Arabidopsis lines. (g) Phenotype of 8-d-old *PbTCP13*-OE seedlings grown on MS medium with 4% glucose (scale bar, 5 mm). (h) Plant height of 8-week-old WT and *PbTCP13*-OE Arabidopsis plants under 4% glucose treatment (scale bar, 5 cm). (i) Expression levels of SCW biosynthetic genes under 4% glucose treatment. (j) Lignin content under glucose-free conditions. (k) Secondary cell wall thickness of vessel cells under 4% glucose treatment. Data are presented as means ± SE (n = 3 for a-f, i, j; n = 13 for k). Significant differences were determined using two-tailed Student's t-test (n.s., no significance, $P > 0.05$; $*P < 0.05$; $**P < 0.01$).

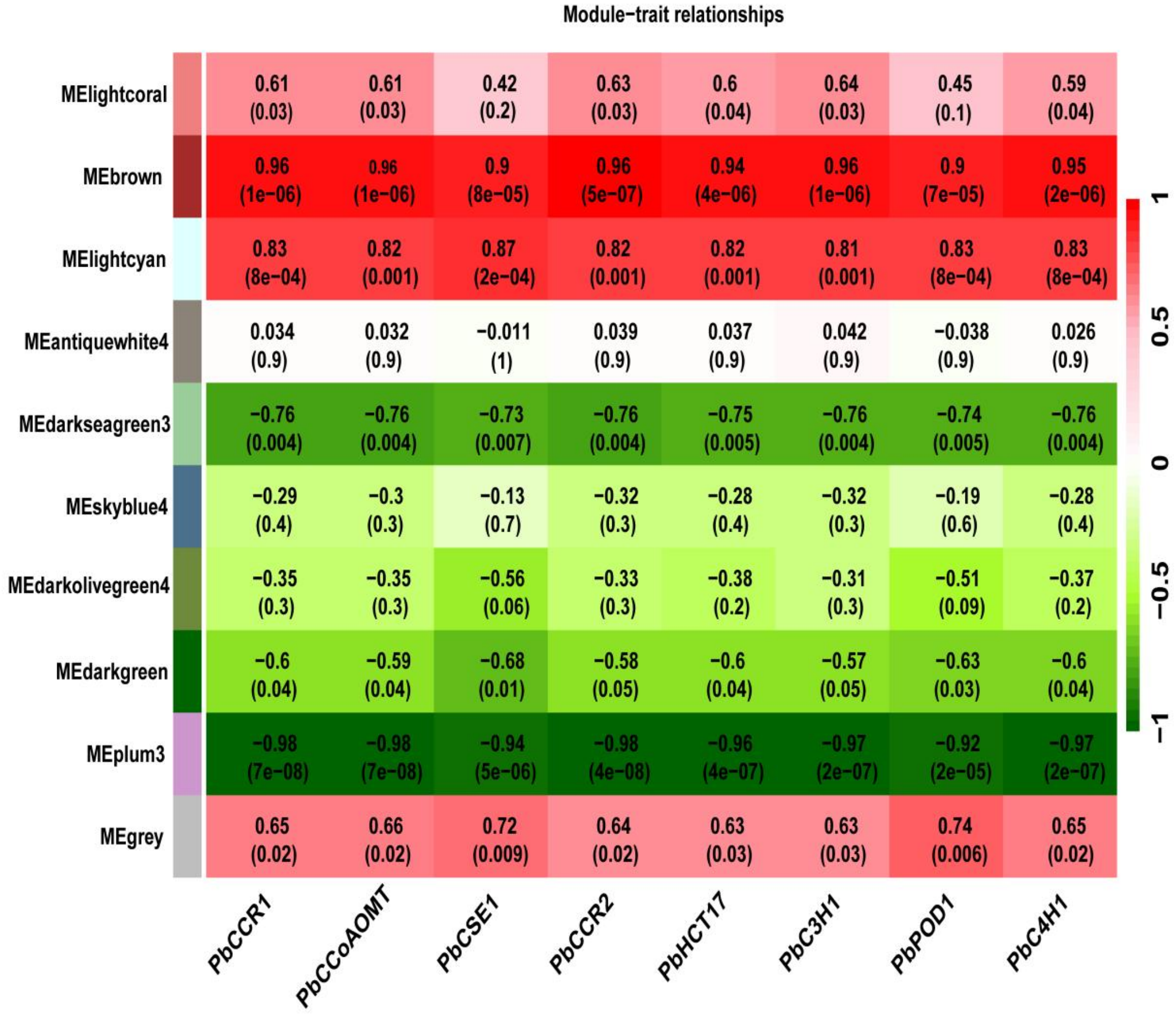

Figure S9. Identification of transcripts by weighted gene co-expression network analysis (WGCNA) of *PbSnRK1*-OE and EV-OE pear calli under 1% or 4% glucose treatment (Supports Figure 4). Module-trait correlations and the corresponding *P*-values are in parentheses. The expression level of lignin-related structural genes functions as trait data. The left panel presents the 10 modules. The color scale on the right presents the module–trait correlations from -1 (green) to 1 (red).

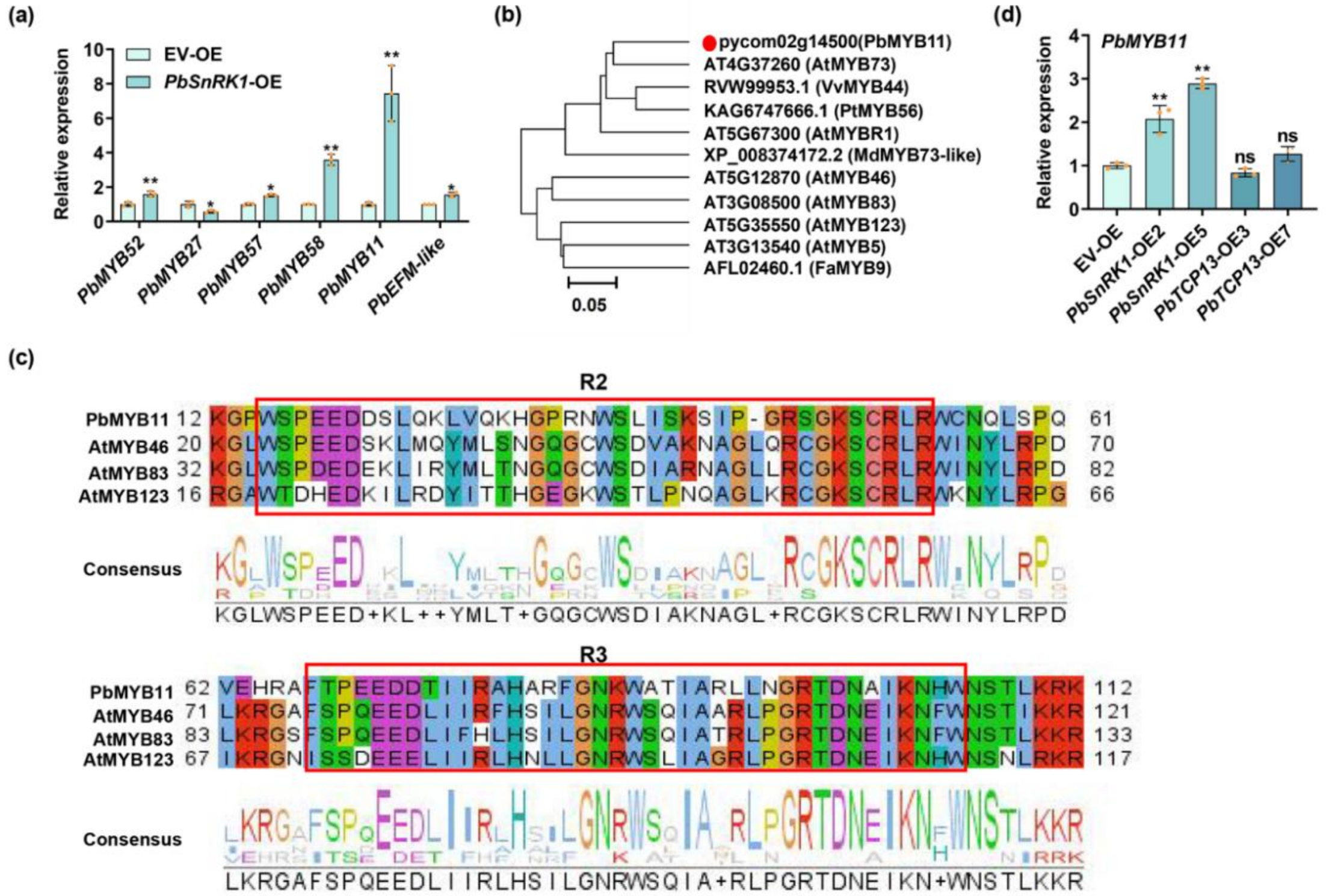

Figure S10 Characterization of *PbMYB11* and its expression pattern (Supports Figure 4). (a) Expression levels of six MYB TFs in the "plum3" module of *PbSnRK1*-OE pear calli under 1% treatment. (b) Phylogenetic analysis of PbMYB11. (c) Amino acid sequence alignment of PbMYB11 with homologous R2R3-MYB genes. The R2R3 DNA-binding domain is highlighted in red boxes. (d) Expression levels of *PbMYB11* in *PbSnRK1*-OE and *PbTCP13*-OE pear calli under 4% glucose treatment. Data are presented as means ± SE (n = 3). Significant differences were determined using two-tailed Student's t-test (n.s., no significance, $P > 0.05$; $^{**}P < 0.01$).

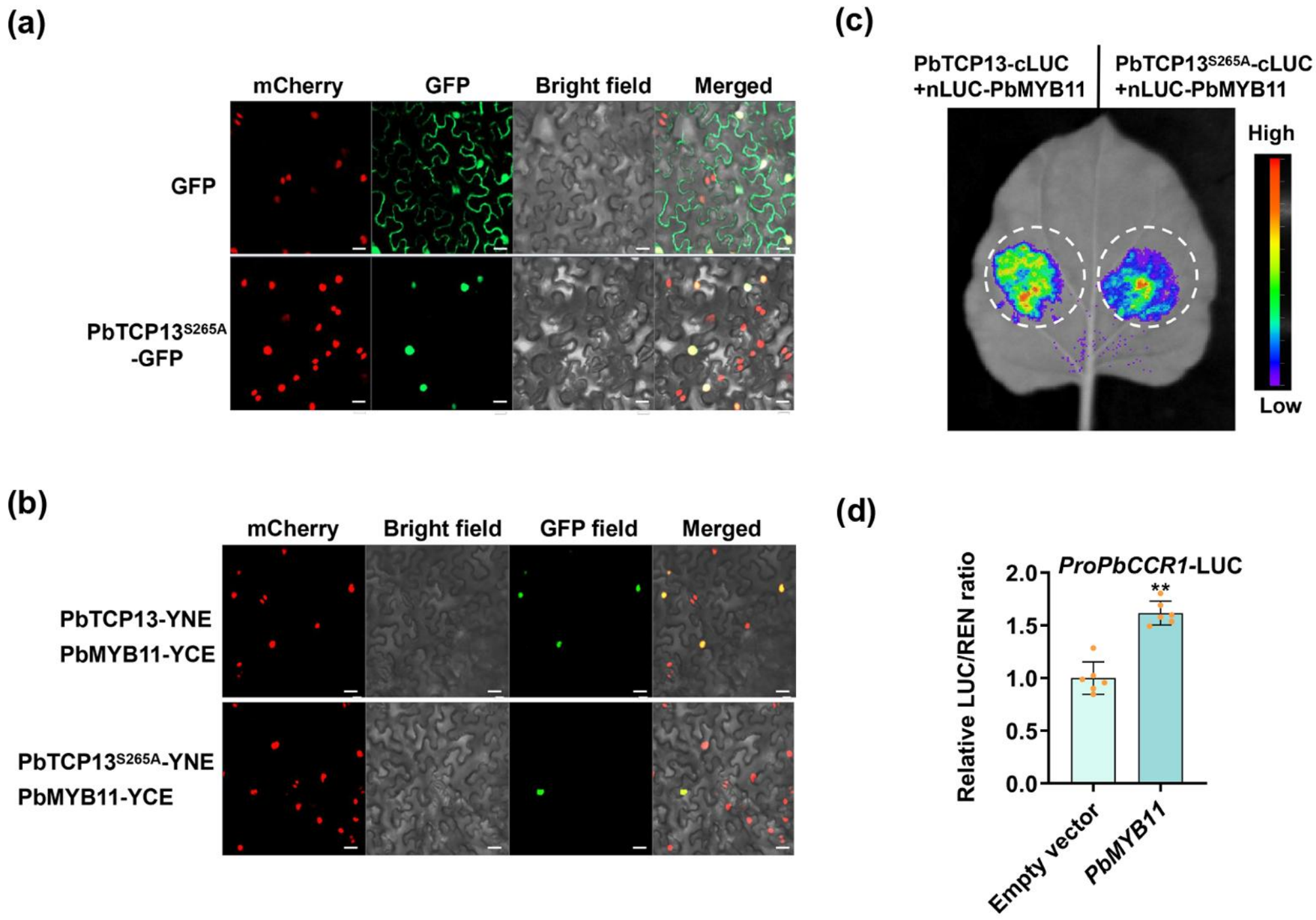

Figure S11 Ser265 phosphorylation regulates PbTCP13-PbMYB11 interaction and *PbMYB11* activation of *PbCCR1* (Supports Figure 4). (a) Subcellular localization showed that PbTCP13^{S265A} was localized in the nucleus of *N. benthamiana* leaf cells (scale bar, 20 μm). (b) BiFC and (c) LCI assays revealed that the S265A mutation in PbTCP13 slightly attenuated the interaction intensity between PbTCP13 and PbMYB11 in *N.benthamiana* leaf cells (scale bar, 20 μm). (d) Dual-luciferase reporter assays verified that *PbMYB11* significantly activates the transcriptional activity of the *PbCCR1* promoter in *N. benthamiana* leaf cells. Data are presented as means ± SE (n = 6). Significant differences were determined using two-tailed Student's t-test (**P < 0.01).

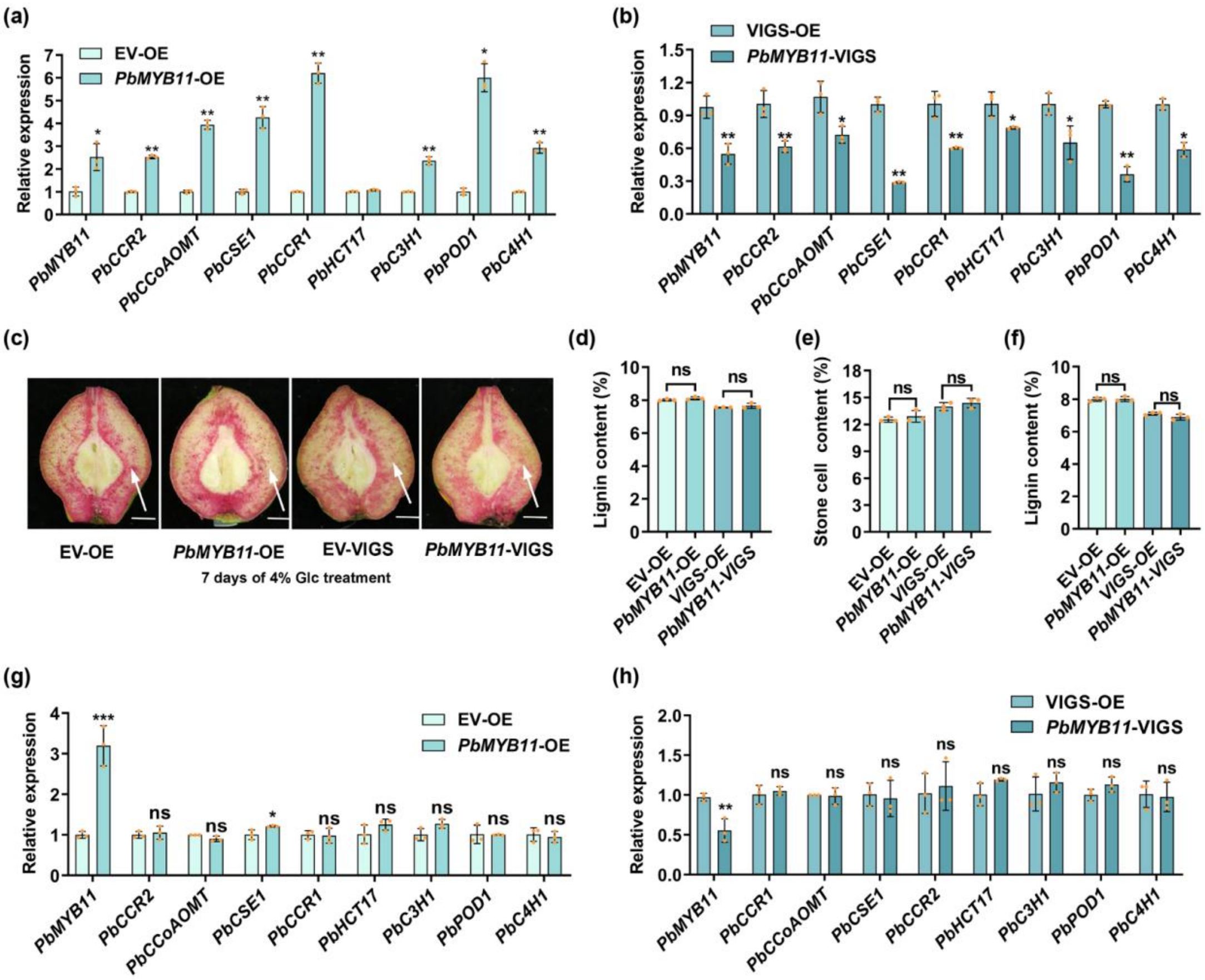

Figure S12 Lignin accumulation and lignin biosynthetic gene expression in *PbMYB11*-OE pear fruits under different glucose treatments (Supports Figure 5). (a,b) Expression levels of lignin biosynthetic genes in pear fruits with transient (a) *PbMYB11*-OE or (b) *PbMYB11*-VIGS under 1% glucose treatment. (c) Phloroglucinol-HCl staining of EV-OE, *PbMYB11*-OE, EV-VIGS, and *PbMYB11*-VIGS transgenic pear fruits following 4% glucose treatment for 7 days (scale bar, 0.5 cm). (d) Lignin content and (e) stone cell content in the above transgenic pear fruits. (f) Lignin content in the above transgenic pear fruits under normal growth conditions (no exogenous glucose). (g,h) RT-qPCR analysis of lignin biosynthetic genes in pear fruits with transient (g) *PbMYB11*-OE or (h) *PbMYB11*-VIGS under 4% glucose treatment. Data are presented as means ± SE (n = 3). Significant differences were determined using two-tailed Student's t-test (n.s., no significance, $P > 0.05$; $*P < 0.05$; $**P < 0.01$).

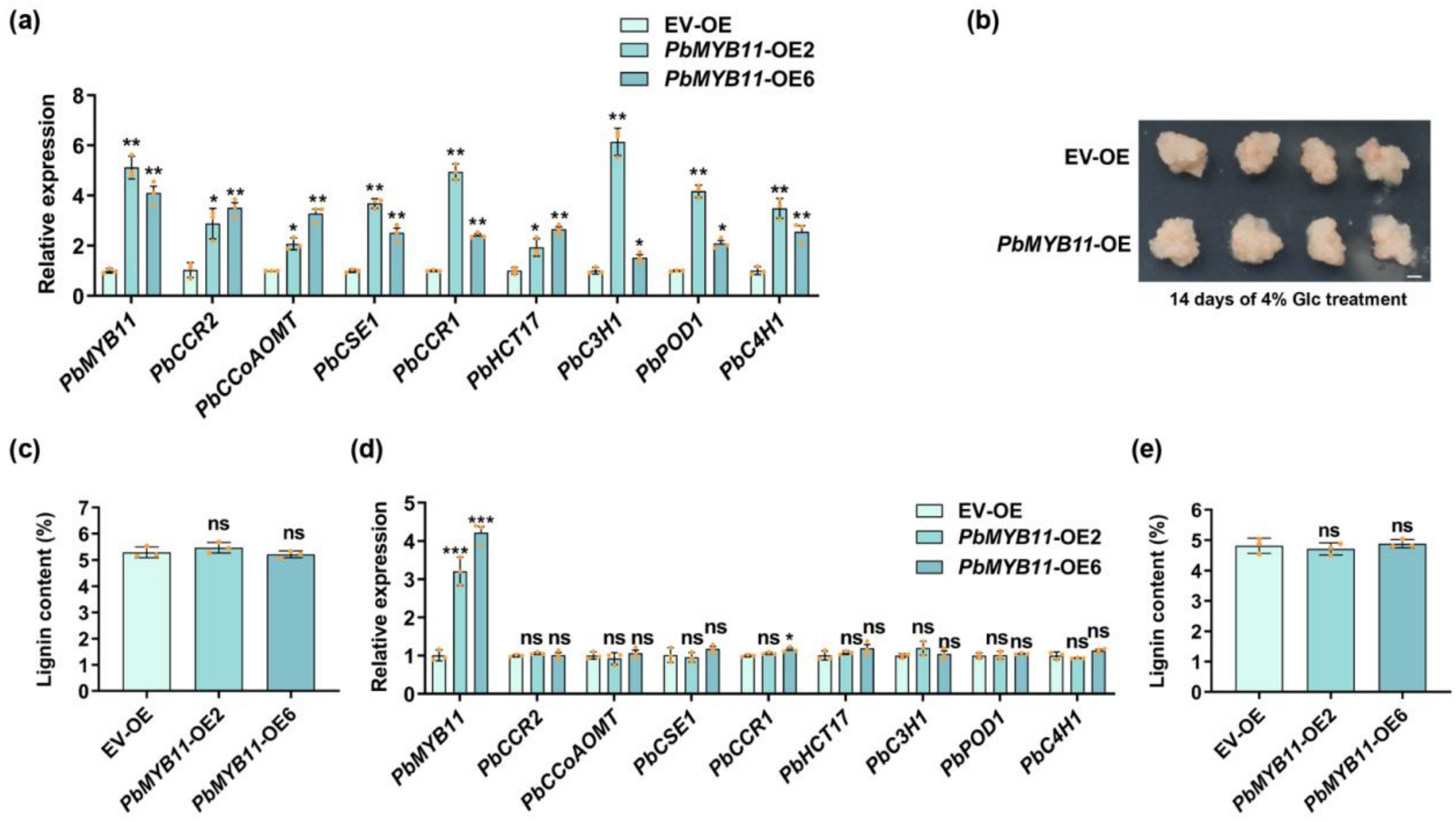

Figure S13 Lignin accumulation and lignin biosynthetic gene expression in *PbMYB11*-OE pear calli under different conditions (Supports Figure 5). (a) Expression levels of lignin biosynthetic genes in EV-OE and *PbMYB11*-OE pear calli under 1% glucose treatment. (b) Phloroglucinol-HCl staining of EV-OE and *PbMYB11*-OE transgenic pear calli following 4% glucose treatment (scale bar, 0.5 cm). (c) Lignin content in the above transgenic pear calli. (d) RT-qPCR analysis of lignin biosynthetic genes in the above transgenic pear calli. (e) Lignin content in EV-OE and *PbMYB11*-OE transgenic pear calli under normal growth conditions (no exogenous glucose). Data are presented as means ± SE (n = 3). Significant differences were determined using two-tailed Student's t-test (n.s., no significance, $P > 0.05$; *$P < 0.05$; **$P < 0.01$).

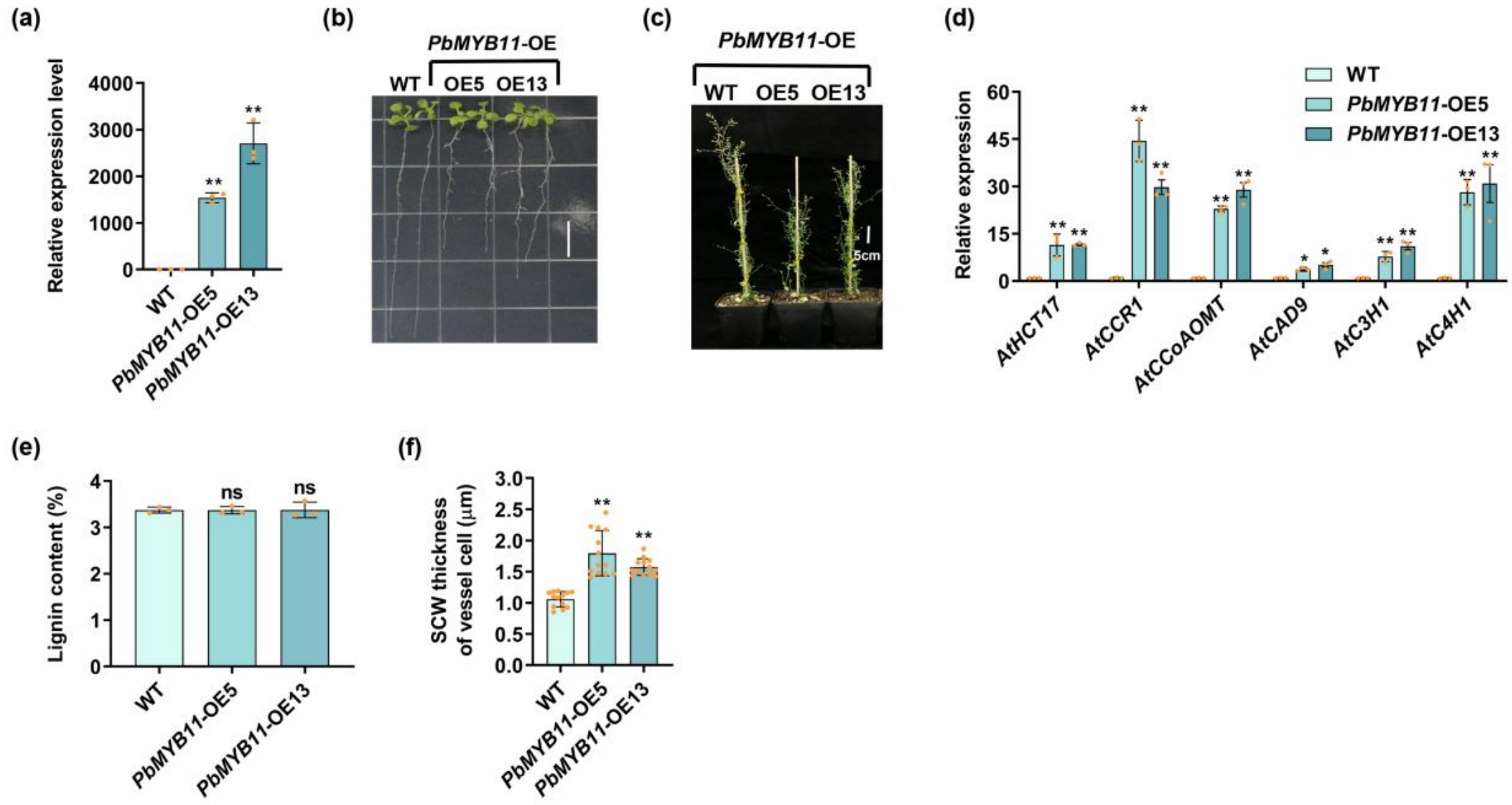

Figure S14 Heterologous expression of *PbMYB11* regulates lignin biosynthesis in Arabidopsis (Supports Figure 5). (a) Expression levels of *PbMYB11* in WT and two independent *PbMYB11*-OE transgenic Arabidopsis lines. (b) Phenotype of 8-d-old *PbMYB11*-OE seedlings grown on MS medium with 1% glucose (scale bar, 5 mm). (c) Plant height of 8-week-old WT and *PbMYB11*-OE Arabidopsis plants under 1% glucose treatment (scale bar, 5 cm). (d) Expression levels of lignin biosynthetic genes under 1% glucose treatment. (e) Lignin content under glucose-free conditions. (f) SCW thickness of vessel cells under 1% glucose treatment. Data are presented as means ± SE (n = 3 for a, d, e; n = 13 for f). Significant differences were determined using two-tailed Student's t-test (n.s., no significance, $P > 0.05$; *$P < 0.05$; **$P < 0.01$).

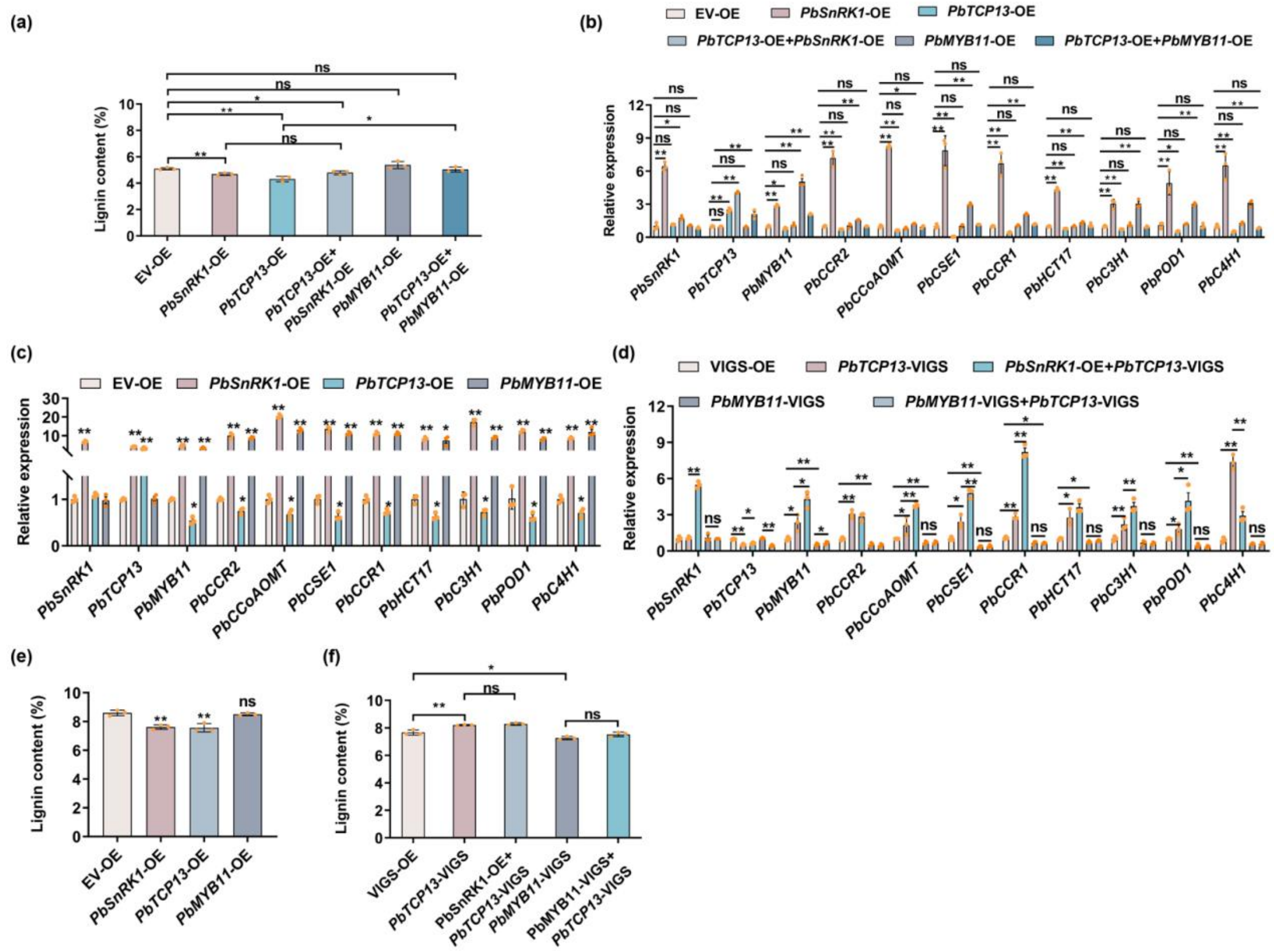

Figure S15 Lignin accumulation and gene expression of the PbSnRK1-PbTCP13-PbMYB11 module under different glucose treatments (Supports Figure 6). (a) Lignin content in EV-OE, *PbSnRK1*-OE, *PbTCP13*-OE, *PbTCP13*-OE/*PbSnRK1*-OE, *PbMYB11*-OE, and *PbTCP13*-OE/*PbMYB11*-OE transgenic pear calli under 4% glucose treatment. (b) RT-qPCR analysis of lignin biosynthetic genes in EV-OE, *PbSnRK1*-OE, *PbTCP13*-OE, *PbTCP13*-OE/*PbSnRK1*-OE, *PbMYB11*-OE, and *PbTCP13*-OE/*PbMYB11*-OE transgenic pear calli under 1% glucose treatment. (c) RT-qPCR analysis of lignin biosynthetic genes in EV-OE, *PbSnRK1*-OE, *PbTCP13*-OE, and *PbMYB11*-OE transgenic pear fruits under 1% glucose treatment. (d) RT-qPCR analysis of lignin biosynthetic genes in EV-VIGS, *PbTCP13*-VIGS, *PbSnRK1*-OE/*PbTCP13*-VIGS, *PbMYB11*-VIGS, and *PbMYB11*-VIGS/*PbTCP13*-VIGS transgenic pear fruits under 1% glucose treatment. (e,f) Lignin content in the above transgenic pear fruit lines under 4% glucose treatment. Data are presented as means ± SE (n = 3). Significant

differences were determined using two-tailed Student's t-test (n.s., no significance, $P > 0.05$; $^{*}P < 0.05$; $^{**}P < 0.01$).
